# Unique features of conventional and nonconventional introns in *Euglena gracilis*

**DOI:** 10.1186/s12864-024-10495-9

**Published:** 2024-06-13

**Authors:** Pingwei Gao, Yali Zhao, Guangjie Xu, Yujie Zhong, Chengfu Sun

**Affiliations:** 1 Scientific Research Center, Chengdu Medical College, Chengdu, 610500 China; 2 Clinical Laboratory Department, Zigong Hospital of Women’s and Children’s Healthcare, Zigong, 643002 China

**Keywords:** Splice site, Outron, SL-RNA, Polypyrimidine tract, Extended U6/5' ss duplex

## Abstract

**Background:**

Nuclear introns in Euglenida have been understudied. This study aimed to investigate nuclear introns in Euglenida by identifying a large number of introns in *Euglena gracilis* (*E. gracilis*), including *cis*-spliced conventional and nonconventional introns, as well as *trans*-spliced outrons. We also examined the sequence characteristics of these introns.

**Results:**

A total of 28,337 introns and 11,921 outrons were identified. Conventional and nonconventional introns have distinct splice site features; the former harbour canonical GT/C-AG splice sites, whereas the latter are capable of forming structured motifs with their terminal sequences. We observed that short introns had a preference for canonical GT-AG introns. Notably, conventional introns and outrons in *E. gracilis* exhibited a distinct cytidine-rich polypyrimidine tract, in contrast to the thymidine-rich tracts observed in other organisms. Furthermore, the SL-RNAs in *E. gracilis*, as well as in other *trans*-splicing species, can form a recently discovered motif called the extended U6/5’ ss duplex with the respective U6s. We also describe a novel type of alternative splicing pattern in *E. gracilis*. The tandem repeat sequences of introns in this protist were determined, and their contents were comparable to those in humans.

**Conclusions:**

Our findings highlight the unique features of *E. gracilis* introns and provide insights into the splicing mechanism of these introns, as well as the genomics and evolution of Euglenida.

**Supplementary Information:**

The online version contains supplementary material available at 10.1186/s12864-024-10495-9.

## Background

Intervening sequences known as introns are present in eukaryotic genomes. These introns need to be removed during pre-mRNA maturation, a process that is catalysed by the spliceosome. The spliceosome is a large ribonucleoprotein complex composed of five small nuclear RNAs (snRNAs), U1, U2, U4, U5 and U6, along with numerous associated proteins [1]. During splicing, specific RNA elements within introns, including the 5’ splice site (ss) and 3’ ss, internal branch point sequence, and polythymidine tract (Py tract), are sequentially recognized by corresponding snRNAs and/or proteins [2].

The canonical ss displays a dinucleotide configuration with 5’ GT and 3’ AG. This configuration is also observed in SL-mediated *trans*-splicing, a process in which a short exon from spliced leader RNA (SL-RNA) is added to pre-mRNAs [3]. In *trans*-splicing, the 5’ GT is located within SL-RNA, while the 3’ AG is found in the 5’ terminal intron region (referred to as the outron) of pre-mRNAs. Additionally, the secondary structure formed within the 5’ ss region of SL-RNA mimics U1/5’ ss base pairing and renders U1 unnecessary for *trans*-splicing [3, 4]. However, whether other RNA motifs that are crucial for *trans*-splicing are present in SL-RNAs remains unclear.

Euglenida, also known as euglenids or euglenoids, exhibit both *cis*- and *trans*-splicing. Interestingly, preliminary studies indicate that this group possesses numerous nonconventional introns, in addition to the canonical or conventional GT-AG type. First described by Tessier *et al*., nonconventional introns lack the GT/AG dinucleotide in one or both ss but contain structured RNA motifs that are capable of terminal base pairing [5–10]. The origin of nonconventional introns is hypothesized to be transposon elements, as found in other species [5, 11, 12]. In accordance with this hypothesis, their splicing is believed to differ from that of conventional introns and may involve the formation of circular rather than lariat RNA molecules [13]. In a recent study, only a few hundred introns, primarily conventional introns, were identified [14]. Thus, a comprehensive characterization of the distribution and properties of conventional and nonconventional introns in Euglenida is still lacking.

In our recent investigation of the splicing system in *Euglena gracilis* (*E. gracilis*), we identified hundreds of spliceosomal proteins, as well as U2 and U6 snRNAs [15]. Notably, the spliceosome in *E. gracilis* (in contrast to its evolutionary cousins Trypanosoma and Leishmania) exhibits relatively high similarity to that of humans. In the present study, we utilised transcriptomic and genomic data to identify more than 28,000 introns in *E. gracilis*. Further characterization of these introns revealed unique features.

## Results and discussion

### Identification of nuclear introns in *E. Gracilis*

We previously conducted third-generation transcriptomic RNA sequencing (RNA-seq) [15]. During data analysis, we detected the presence of sequences originating from the chloroplast and mitochondrial genomes, as well as nuclear rDNA genes. These sequences were considered “contaminated” and were subsequently removed, leaving only sequences comprising nuclear pre-mRNA/mRNA. After removing redundancy with cd-hit [16], a total of 18,643 sequences were obtained and used as the starting point for intron identification.

**Fig. 1:**
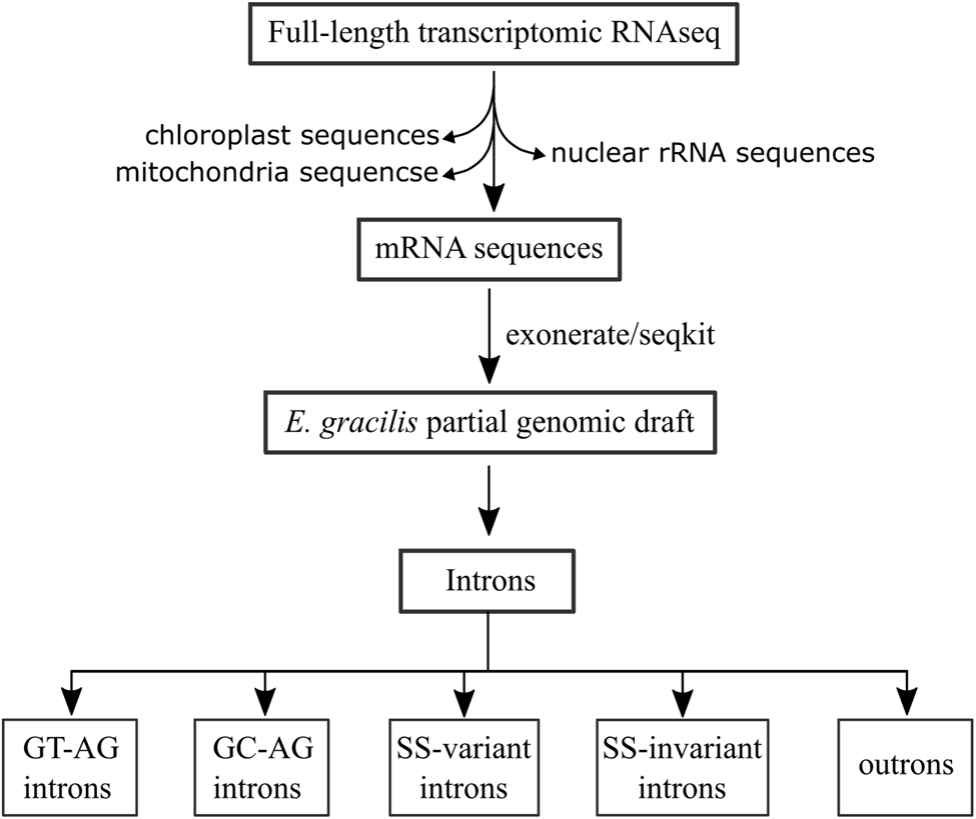
Working procedure used to identify nuclear introns in *E. gracilis*

We utilised the Exonerate program [17] to align the mRNA sequences and genomic draft data [14]. The identified intron sequences were subsequently extracted (Fig. 1). We found that the default settings of exonerate were effective in identifying canonical GT-AG introns but were inadequate for identifying nonconventional introns. To overcome this limitation, a custom position-specific score matrix (PSSM) was employed in our analysis. With the use of this PSSM, nonconventional introns were accurately annotated (Fig. S1). However, we observed that a few conventional introns were misannotated despite the vast majority being correctly identified. To address this issue, we manually inspected all introns identified by Exonerate.

The identified introns were further categorized into four groups: GT-AG, GC-AG, ss-invariant and ss-variant (Fig. 1). The GT-AG group is the conventional type, while the GC-AG group is a variation of that type. The remaining two groups were characterized by terminal dinucleotides that were neither GT-AG nor GC-AG. Moreover, the ss-variant group refers to introns with terminal nucleotides that had multiple possibilities and could not be definitively determined (Fig. S2). Due to the absence of the canonical GT/GC-AG dinucleotide on one or both termini, the ss-invariant and ss-variant introns were classified as nonconventional introns in this study.

Additionally, we determined the 3’ terminal region of the outrons (Fig. 1). Initially, mRNA sequences containing the SL exon were extracted. Subsequently, the SL exon was replaced with the AG dinucleotide. These AG-attached sequences were then searched against genome sequences using BLAST. Finally, genomic sequences that had hits were identified, and the corresponding outron regions were extracted with seqkit [18].

### Overview of nuclear introns in *E. Gracilis*

**Fig. 2:**
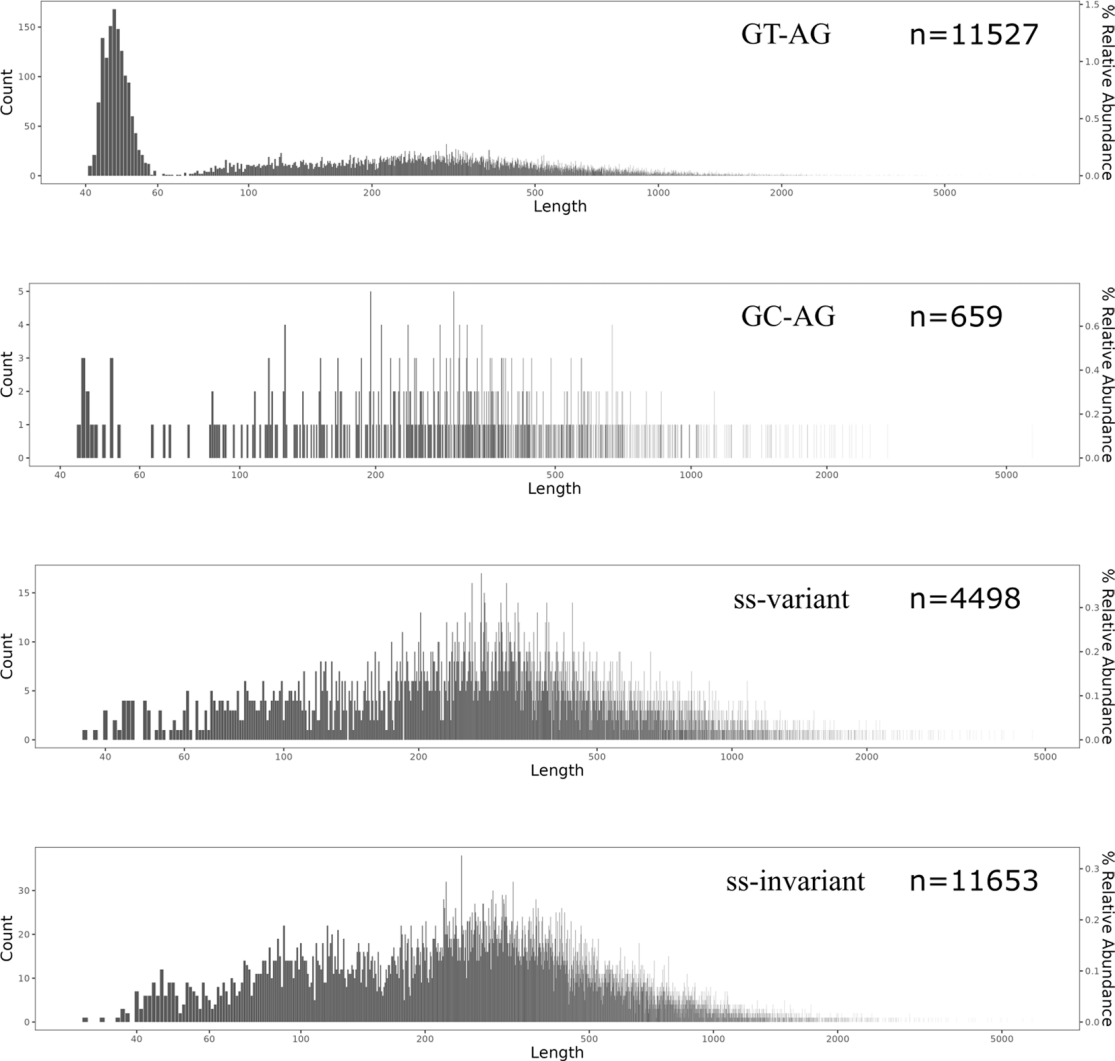
Length distribution of nuclear introns in *E. gracilis*. Intron groups (GT-AG, GC-AG, ss-invariant, and ss-variant) and their respective numbers are labelled in each panel

A total of 28,337 introns were identified. The sequence statistics of all four groups of introns (GT-AG, GC-AG, ss-invariant, and ss-variant) are summarized in Table 1. The length distribution of introns in all four groups was similar, with minimal lengths less than 50 base pairs (bp) and maximal lengths ranging from 4,682 bp to 8,197 bp. Notably, there was a prominent peak of short introns with a sequence length less than 60 bp in the GT-AG group (Fig. 2). Specifically, the percentages of short introns were 9.82% (1332 out of 11,527) for GT-AG introns, 1.37% (13 out of 659) for GC-AG introns, 0.89% (40 out of 4495) for ss-invariant introns, and 1.25% (146 out of 11,656) for ss-variant introns. Furthermore, the distribution profiles of ss-invariant and ss-variant introns, both of which are nonconventional introns, were highly similar, indicating putative shared features between these two groups of introns. Additionally, all four groups exhibited similar average lengths (Table 1).

**Table 1:** Statistics of *E. gracilis* nuclear introns

Type	number	min_len	avg_len	max_len
GT-AG	11,527	41	512.5	8197
GC-AG	659	44	522	5701
ss-invariant ss-variant	4498 11,653	36 30	538.8 516.8	4682 5931

In addition to the *cis*-splicing introns mentioned above, a total of 11,921 outrons were identified. As the transcription start sites of these outrons are currently unknown, their lengths were not determined. For subsequent analysis, we focused solely on the 20-bp long 3’ terminal region of the outrons.

### The 5’ ss region of conventional nuclear introns differs from that of SL-RNAs

**Fig. 3:**
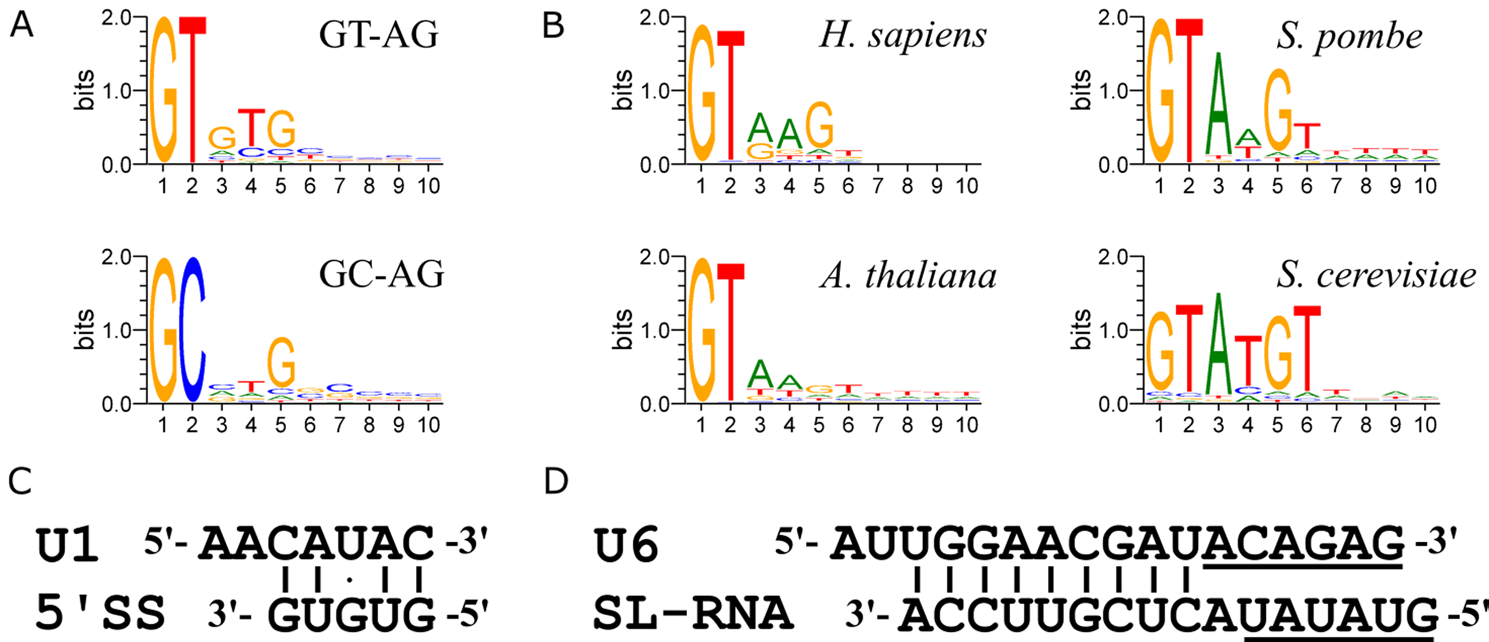
Sequence conservation of the 5’ ss region of conventional introns. (**A**) Sequence conservation of the 5’ ss region of the GT-AG and GC-AG introns in *E. gracilis*. (**B**) Sequence conservation of the 5’ ss region of introns in humans, *A. thaliana*, *S. pombe*, and *S. cerevisiae*. (**C**) Base pairing between the consensus sequence motif of the 5’ ss of GT-AG introns and U1 in *E. gracilis*. (**D**) Extensive base pairing between sequences upstream of the U6 ACAGA box and downstream of the 5’ ss of SL-RNA in *E. gracilis*. The ACAGA box of U6 and the 5’ ss of SL-RNA are underlined. All sequence logos are plotted on a vertical scale with 0–2 bits of information

We initially examined the conservation of the 10-bp 5’ ss region in the GT-AG and GC-AG introns. The consensus sequence for the GT-AG introns was GTGTG (Fig. 3A). In the GC-AG introns, only guanine (G) at the fifth position was prominent (Fig. 3A). To compare these findings with those of other species, we retrieved U2-type introns from humans, *Arabidopsis thaliana* (*A. thaliana*), *Schizosaccharomyces pombe* (*S. pombe*) and *Saccharomyces cerevisiae* (*S. cerevisiae*) from the Intron Annotation and Orthology Database (IAOD) and analysed their 5’ ss sequences (Fig. 3B). Our results for these four species agree well with those of previous studies [19–21]. The prominent G at the fifth position is also present in humans and yeasts, which belong to the Opisthokonta clade. However, this G is not prominent in *A. thaliana*.

Next, we examined the base pairing between the consensus GTGTG motif in GT-AG introns and the 5’ terminal region of *E. gracilis* U1 (Fig. 3C). All five bases in the intron motif were involved in base pairing with U1, including a G•U wobble base pair, as previously suggested for a few genes [9, 13, 22]. This finding suggested that U1 is likely to recognize this motif effectively during splicing. Similarly, substantial internal base pairing was observed in the corresponding 5’ ss region of SL-RNA. The 5’ ss sequence of SL-RNA is GTATA, and differs from the consensus intron motif by two Gs. Notably, only a few introns (39 out of 11,527) in the GT-AG group possessed the 5’ ss sequence of SL-RNA. This result indicates that the 5’ ss in GT-AG and SL-RNA exhibit distinct base compositions and may function specifically in *cis*- and *trans*-splicing, respectively.

### Conservation of the extended U6/5’ ss duplex in SL-mediated *trans*-splicing species

The presence of extensive internal base pairing in the 5’ ss region of *E. gracilis* SL-RNA led us to compare other base pairing structures between SL-RNA and other snRNAs. Upon aligning the 5’ ss region of SL-RNA with U6, we observed another significant base pairing between the downstream region of the 5’ ss and the upstream region of the U6 ACAGA box (Fig. 3D). This U6/SL-RNA helix region is also termed the extended U6/5’ ss helix, which was initially identified in the cryogenic electron microscopy (cryo-EM) structure of a precatalytic human spliceosomal B complex [23]. However, unlike the human version, which harbours five inconsecutive base pairs, the *E. gracilis* U6/SL-RNA helix consisted of eight consecutive base pairs, suggesting a strong interaction between the two moieties.

We asked whether the U6/SL-RNA helix is also present in other species that undergo SL-mediated *trans*-splicing. Alignment of *Caenorhabditis elegans* (*C. elegans*) U6 and SL-RNA showed that they also formed eight consecutive base pairs (Fig. S3A). Additionally, U6s and SL-RNAs in *Trypanosoma brucei* (*T. brucei*) and *Leishmania donovani* (*L. donovani*), which are parasites belonging to the Excavate clade, similar to *E. gracilis*, also formed extensive base pairs in this region (Fig. S3A). In summary, we conclude that the formation of the extended U6/5’ ss duplex is a significant feature in species with SL-mediated *trans*-splicing.

Considering that nuclear introns exhibit various base compositions downstream of the 5’ ss, we next asked whether an extended U6/5’ ss helix is present between U6 and certain introns. Upon examining the 10-bp region downstream of the hexanucleotide 5’ ss in humans, *A. thaliana*, *S. pombe*, and *S. cerevisiae*, along with their respective U6 sequences, we identified introns that could form the extended U6/5’ ss helix in these species (Fig. S3B). For instance, the Mei4 intron in *S. cerevisiae* could form five base pairs with U6 in this region, while the second intron of the AT2G30650 gene in *A. thaliana* is capable of forming nine base pairs with U6. This finding suggested that the formation of the extended U6/5’ ss helix is dependent on the sequence of the intron and not a metazoan-specific signature, as proposed previously [23]. Therefore, this duplex likely appears in most species as long as the intron has the ability to form this motif with U6. Three proteins in the spliceosomal B complex surround this duplex [24]. Among these proteins, Prp38 and ZMAT2/Snu23 have homologues in *E. gracilis* [15]. We propose that these proteins may interact with the deeply conserved duplex in *E. gracilis* in a manner similar to that seen in the human spliceosome.

## **The 3’ ss region of conventional nuclear introns possesses a rare C-rich py tract**

**Fig. 4:**
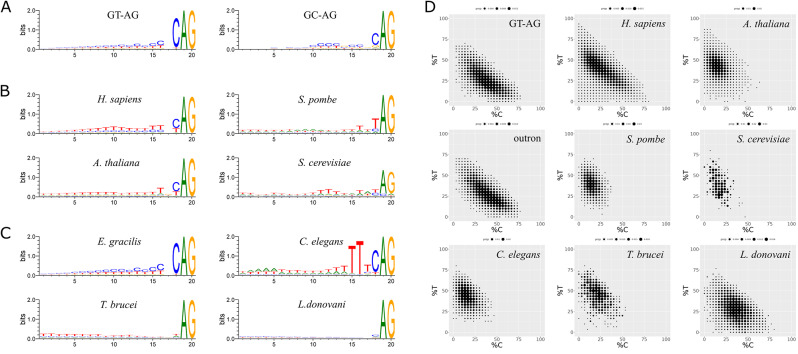
Sequence conservation and composition of the 3’ ss region of conventional introns. (**A**) Sequence conservation of the *E. gracilis* 3’ ss regions of the GT-AG and GC-AG introns. (**B**) Sequence conservation of the 3’ ss regions of introns in humans, *A. thaliana*, *S. pombe*, and *S. cerevisiae*. (**C**) Sequence conservation of the 3’ ss regions of outrons in *E. gracilis*, *C. elegans*, *T. brucei*, and *L. donovani*. (**D**) Ratios of C/T in the 3’ ss regions of introns and/or outrons in E. gracilis, humans, *A. thaliana*, *S. pombe*, *S. cerevisiae*, *C. elegans*, *T. brucei*, and *L. donovani*. All sequence logos are plotted on a vertical scale with 0–2 bits of information

We examined sequence conservation in the 20-bp 3’ ss region of the GT-AG and GC-AG introns (Fig. 4A). Both groups of introns show a similar pattern in this region. Notably, in *E. gracilis*, there is an abundance of cytidine (C) compared to thymidine (T) in the Py tract preceding the 3’ ss. Conversely, when analysing the same 20-bp 3’ ss region in species such as humans, *A. thaliana*, *S. pombe*, and *S. cerevisiae*, a predominant T-rich composition was observed, as previously described [19–21] (Fig. 4B).

Further examination of the sequence conservation in this 3’ ss region was conducted using our identified *E. gracilis* outrons. We found that the aforementioned patterns in *cis* introns, including the C-rich Py tract and the 3’ ss consensus motif CAG, also apply to outrons in *E. gracilis* (Fig. 4C). To compare the outron patterns, we identified the 3’ ss region of outrons in other species that undergo *trans*-splicing. Through analysis of the respective transcriptomic and genomic sequences, we obtained a total of 4,535, 976 and 12,030 outrons from *C. elegans* (SL1 only), *T. brucei*, and *L. donovani*, respectively. Interestingly, the outron patterns from different species exhibited clear distinct features. For instance, *C. elegans* had an adenosine (A)/T-rich Py tract and a unique 3’ ss consensus motif of the hexanucleotide TTTCAG, which has been previously characterized in a few outrons [25]. In the case of *T. brucei* and *L. donovani*, we found a noticeable difference in the Py tract region, with the former being slightly more T-rich (as previously reported [26, 27]) than the latter. Notably, the slightly C-rich pattern in the outrons of *L. donovani* was also seen in its conventional introns, several genes of which have been examined previously [28].

To gain an overview of the base ratio (T vs. C) in the Py tract, we plotted the distribution of these two bases in introns across all the aforementioned species (Fig. 4D). The results revealed that the majority of *E. gracilis* introns (GT-AG introns and outrons) were C-rich, while introns in other species, except for *L. donovani*, demonstrated a predominant T-rich composition. In *L. donovani*, most outrons exhibited only a very weak tendency towards a C-rich composition. In conclusion, these findings suggest that *E. gracilis* possesses a unique base composition in the Py tract. The presence of the C-rich Py tract in *E. gracilis*, which has not been extensively characterized in other species to date, suggests a novel pattern of recognition by its cognate splicing factor, U2AF2. In addition, the high similarity in the patterns of the 3’ ss region between conventional *cis* introns and outrons in *E. gracilis* indicates that their splicing reactions are dependent on the spliceosome.

### Nonconventional nuclear introns harbour structured terminal base pairing motifs

As mentioned earlier, ss-invariant and ss-variant introns, which are considered nonconventional introns, show similar length distributions. However, the sequence conservation of nonconventional introns has only been examined in a limited number of genes. In this study, we aimed to revisit this question using a large number of introns.

**Fig. 5:**
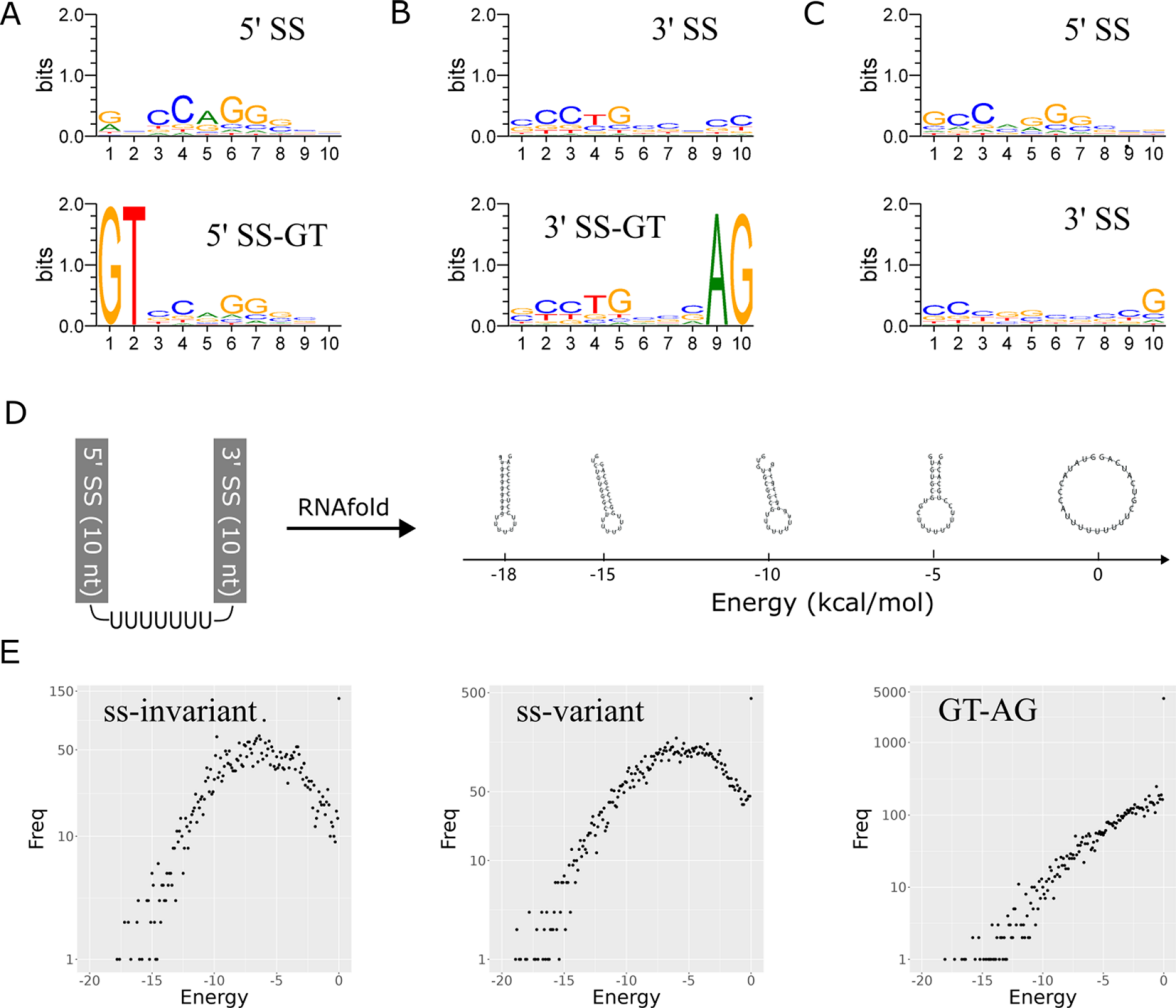
Sequence conservation and base pairing of the ss region of *E. gracilis* nonconventional introns. (**A**) Sequence conservation of the 5’ ss region of nonconventional ss-invariant introns. (**B**) Sequence conservation of the 3’ ss region of nonconventional ss-invariant introns. (**C**) Sequence conservation of the 5’ and 3’ ss regions of nonconventional ss-variant introns. (**D**) Illustration of terminal base pairing generated by RNAfold and the energies associated with different levels of base pairing. (**E**) Plots of the energy and frequency of terminal base pairing of ss-invariant, ss-variant and GT-AG introns in *E. gracilis*. All sequence logos are plotted on a vertical scale with 0–2 bits of information

Initially, we examined the 10-bp 5’ ss region of ss-invariant introns. We identified a CCAGG pentanucleotide consensus sequence spanning from the + 3 to + 7 positions in this group of introns (Fig. 5A). Furthermore, we specifically extracted ss-invariant introns with a 5’ terminal GT dinucleotide and plotted their sequence conservation. Once again, they displayed a similar consensus sequence (Fig. 5A). Moving on to the 10-bp 3’ ss region, we observed a CCTG tetranucleotide consensus sequence spanning from positions − 9 to -6 (Fig. 5B). This consensus sequence was still present in ss-invariant introns with a 3’ terminal AG dinucleotide. Finally, we examined the ss-variant introns (Fig. 5C). In terms of the 5’ ss region, the sequence conservation of ss-variant introns was similar to that of ss-invariant introns. However, there was a slight difference in the sequence conservation of the 3’ ss regions between ss-invariant and ss-variant introns. As the terminal nucleotides of ss-variant introns cannot be determined with certainty, the division of ss-invariant and ss-variant introns is driven solely by technical considerations. Therefore, importantly, our analysis results may underrepresent sequence conservation in ss-variant introns due to the annotation of intron borders by Exonerate.

The consensus sequences observed at both termini suggest the possibility of base pairing between the two ends, as previously suggested for a few genes [5–7]. To investigate the potential differences in terminal base pairing between ss-invariant and ss-variant introns and compare them to canonical GT-AG introns, we examined the secondary RNA structure formed between the 5’ and 3’ terminal regions using RNAfold. As depicted in Fig. 5D, the terminal 10-bp sequences were ligated with seven uridines (U) for looping, resulting in a chimeric sequence 27 bp in length. The degree of base pairing in this chimeric sequence was reflected by the energy value (Fig. 5D). By plotting the energy and frequency of introns in the ss-invariant, ss-variant and GT-AG groups, we discovered that ss-invariant and ss-variant introns exhibited similar patterns, with the majority of introns displaying terminal base pairing within an energy range of -10 to -2 kcal/mol (Fig. 5E). In contrast, GT-AG introns displayed a rough linear distribution without any distinct peak (Fig. 5E). Additionally, the percentages of introns with zero kcal/mol of energy relative to the total number of introns were 2.91% (131 out of 4498), 3.74% (436 out of 11,653), and 35.35% (4075 out of 11,527) for ss-invariant, ss-variant, and GT-AG introns, respectively. This result indicates that nonconventional introns can undergo terminal base pairing, while conventional introns cannot. This finding strongly suggests that, in comparison to conventional introns, a divergent splicing mechanism that is presumably spliceosome independent is utilised by nonconventional introns. Nevertheless, as the splicing of nonconventional introns could occur in either a spliceosome-independent or spliceosome-dependent manner in other species [11, 12], experimental verification is required to confirm this trend in *E. gracilis*.

### Novel alternative splicing patterns in *E. Gracilis* nuclear introns

With the *E. gracilis* nuclear introns in hand, we explored the alternative splicing (AS) pattern of *E. gracilis* nuclear genes. To this end, a BLAST search was conducted on the 28,337 identified introns against the 18,643 mRNA sequences, and only the first hit of the subject sequence was extracted, followed by manual examination to exclude any putative paralogous genes.

**Fig. 6:**
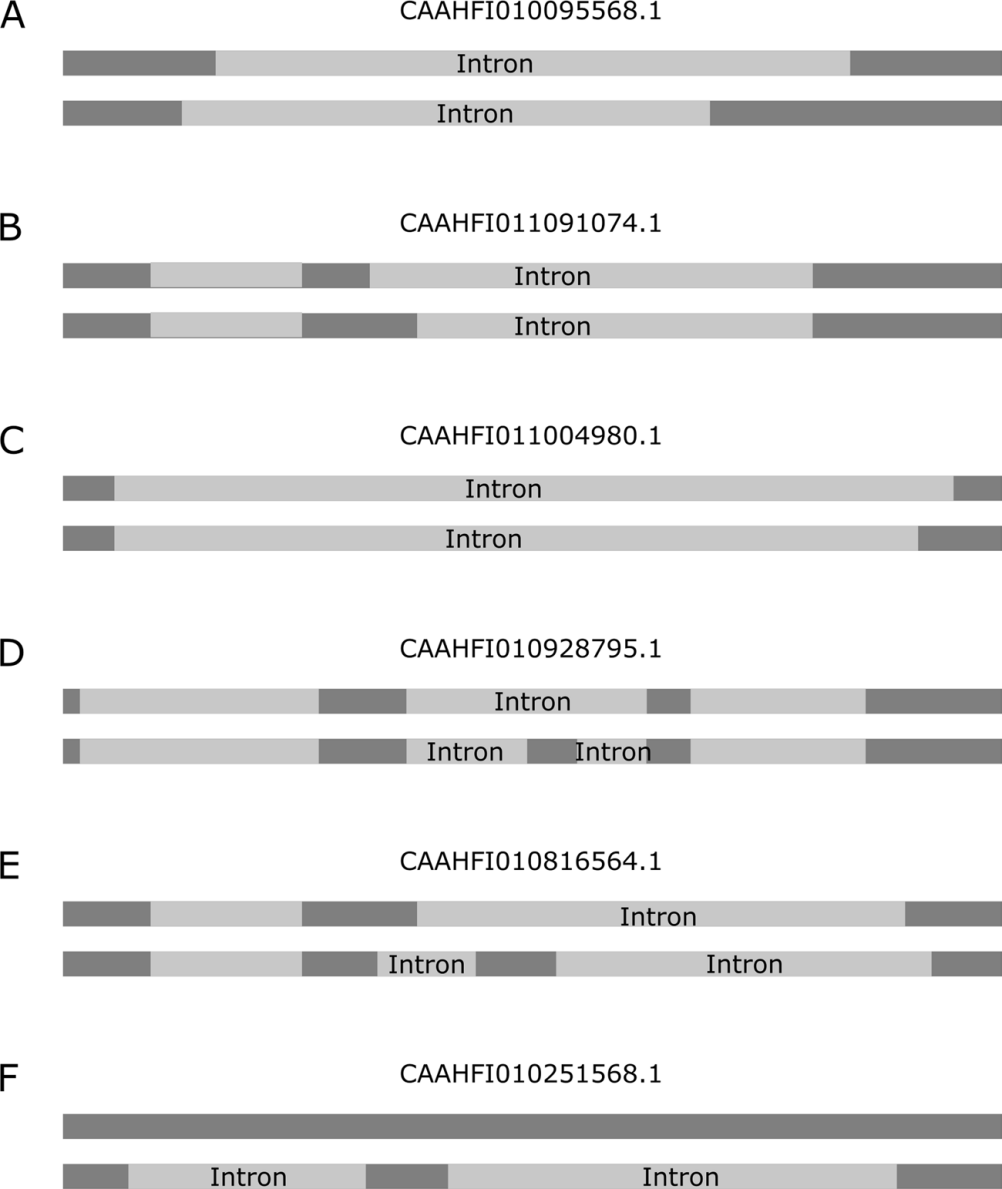
AS patterns of *E. gracilis* nuclear introns. Schematic examples of overlapping (**A**), A5SS (**B**), A3SS (**C**), SE (**D**), atypical SE (**E**), and RI (**F**) are shown. Exons are depicted in dark grey, and introns are depicted in light grey. The genomic accessions for each pattern are indicated above the transcripts, and introns are labelled on the transcripts

In total, we identified nine alternative 5’ ss (A5SS), nine alternative 3’ ss (A3SS), 11 skipped exons (SEs) including ten atypical SEs and one canonical SE, and 161 retained introns (RIs) (Fig. 6 and Table S1). Notably, out of the 11 SE events, we found only one canonical SE event. The remaining ten SE events, which we refer to as atypical SEs, involve different locations of terminal ss (Fig. 6). Among these ten atypical SE events, only three had identical 5’ ss locations, and none had identical 3’ ss locations.

In addition to the aforementioned types of AS, we observed that certain pairs of introns overlapped in their locations in the genome but had different terminal ss locations, which we referred to as overlapping for this type of AS (Fig. 6). We identified a total of 59 overlapping AS events (Table S1). Even the atypical SE pattern in *E. gracilis* can be seen as a combination of overlapping to some extent. Thus, this type of AS may interfere with the maturation and function of the coding protein, as codons may shift during translation for some transcripts subject to overlapping splicing (Fig. S4). As the complete genome of *E. gracilis* is currently incomplete, the extent and quantity of this type of AS in *E. gracilis* are not fully understood. Additionally, whether this overlapping AS occurs in other species remains unclear.

### Tandem repeats in *E. Gracilis* nuclear introns are comparable to those in humans

The *E. gracilis* genome has been reported to contain extensive repeat sequences, and some *E. gracilis* genes also contain tandem repeats (TRs) within their introns. For example, the gamma-tubulin gene in *E. gracilis* has an 88-bp GT/GC in its eleventh intron and a 482-bp AT/AC TR in its thirteenth intron. While processing the identified intron sequences in our study, we also observed such TRs in these introns.

**Fig. 7:**
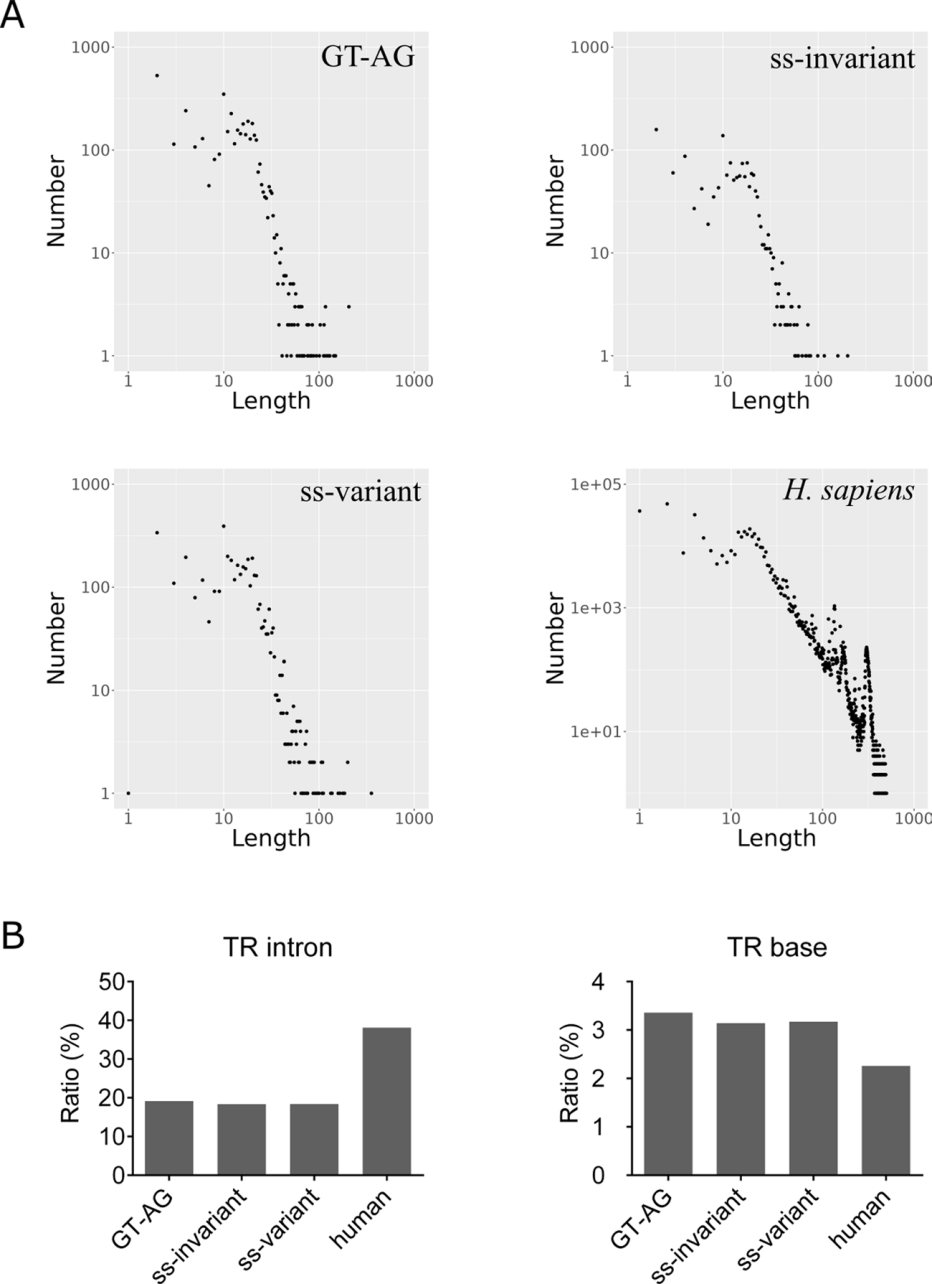
TRs in the nuclear introns of *E. gracilis*. (**A**) The distribution of TR lengths and numbers in GT-AG, ss-invariant and ss-variant introns of *E. gracilis*. The distribution of TRs in human introns is included for comparison. (**B**) Ratios of TRs containing introns and TRs containing bases

To provide a comprehensive overview of TRs in *E. gracilis* introns, we subjected GT-AG, ss-invariant, and ss-variant introns, along with human introns, to TRF, which is a TR finder program [29]. The relationships between the number and length of these TRs were subsequently plotted. The results showed that all these introns displayed similar patterns, with many having short repeats and few having long repeats (Fig. 7A). Additionally, we calculated the ratios of intron number and base content of the TRs (Fig. 7B). We found that the ratio of TR-containing introns was slightly greater in humans than in *E. gracilis*, which may be attributed to the larger size of human introns (with an average length of 6,293.3 bp) when compared to *E. gracilis* introns (which are slightly greater than 500 bp on average, as shown in Table 1). We found that the percentage of TR-containing bases was slightly greater in *E. gracilis* (with an average of 3.22%) than in humans (2.26%) (Fig. 7B). Overall, the TR analysis suggested that *E. gracilis* introns exhibit similar patterns and comparable contents to human introns.

## Summary

In this study, we conducted a large-scale bioinformatic analysis using transcriptomic and genomic data and identified a total of 28,337 *cis* introns and 11,921 outrons in *E. gracilis*. Notably, the intron numbers we reported here may be an underrepresentation, as our research utilised an incomplete and fragmented genome. Our studies confirmed the sequence conservation and identified novel features of nuclear introns in *E. gracilis*, including a unique C-rich Py tract, the presence of an extended U6/5’ ss duplex helix for *trans*-splicing, and a novel AS pattern; these results shed light on the unique aspects of splicing in *E. gracilis*. Further investigation of this remarkable organism will provide insights into novel splicing mechanisms and improve our understanding of intron evolution and comparative genomics in Euglenida.

## Materials and methods

### Intron identification

Third-generation transcriptomic RNA sequencing (RNA-seq) data, with the accession number PRJNA913467 in the NCBI SRA database, were utilised for intron identification analysis. Initially, these data were examined for chloroplast [30], mitochondrial [31], and nuclear rDNA [32] sequences using the BLAST program (version 2.13.0+) [33]. Any sequences that matched these categories were removed from the RNA-seq data. Redundant sequences were then eliminated using cd-hit (version 4.8.1) [16], resulting in the identification of nuclear mRNA sequences, which were subsequently subjected to further analysis. The Exonerate program (version 2.4.0 [17]), was used to align the mRNA sequences with the *E. gracilis* draft genome sequences [14]. A custom PSSM was created using adjusted ratios of A/C/G/T (10/10/50/30 for 5’ ss and 30/10/50/10 for 3’ ss). Subsequently, introns were extracted from the output of Exonerate using a Perl script (github.com/hyphaltip/genome-scripts). Finally, the extracted introns were manually reviewed and categorized into four groups: GT-AG, GC-AG, ss-invariant, and ss-variant.

For outron identification in *E. gracilis*, the first 200-bp sequences containing the SL exon were extracted from the nuclear mRNA sequences. After replacing the *E. gracilis* SL exon with the dinucleotide AG, a BLAST search was conducted against the genome sequences. The resulting outrons were extracted with the seqkit toolkit (version 2.1.0) [18]. This same procedure was also applied for outron identification in *C. elegans* (SL1 only), *T. brucei*, and *L. donovani*, utilising their respective SL exons, RNA-seq datasets (SRR26536558-73 for *C. elegans*, SRR15913764-72 for *T. brucei*, and SRR5272520-7 for *L. donovani*) and genomes.

### Sequence manipulation

The Seqkit toolkit was utilised for various sequence analyses, including subsequence extraction, sequence composition and statistics, and redundancy removal [18]. The relative frequency of nucleotides in terminal ss regions was determined using Weblogo3 [34]. RNA secondary analysis was performed using RNAfold [35]. Tandem repeat analysis was conducted using the TRF program [29]. Sequence plotting analysis was carried out using R (version 4.1.2) [36]. Graphs depicting the intron number and base content analyses of tandem sequences were generated using PRISM 7 (GraphPad, Boston, MA, USA).

### Electronic supplementary material

Below is the link to the electronic supplementary material.

Supplementary Material 1

## Data Availability

All the data generated or analysed during this study are included in the published article.

## Abbreviations

AS: Alternative splicing
cryo-EM: cryogenic electron microscopy
PSSM: Position-specific score matrix
Py tract: Polythymidine tract
RI: Retained intron
SE: Skipped exon
SL: Spliced leader
TR: Tandem repeat
