# Supplementary material for: Unique features of conventional and nonconventional introns in Euglena gracilis
Source: BMC Genomics. 2024 Jun 13;25:595. doi: 10.1186/s12864-024-10495-9 (PMC11170887; doi:10.1186/s12864-024-10495-9)
Supplement: Supplementary file 1 — Supplementary Material 1 [file 12864_2024_10495_MOESM1_ESM.docx]

Supporting information for

**Unique features of conventional and nonconventional introns in *Euglena gracilis***

Pingwei Gao, Yali Zhao, Guangjie Xu, Yujie Zhong and Chengfu Sun

This file includes:

Supplementary Figure S1

Supplementary Figure S2

Supplementary Figure S3

Supplementary Figure S4

Supplementary Table S1


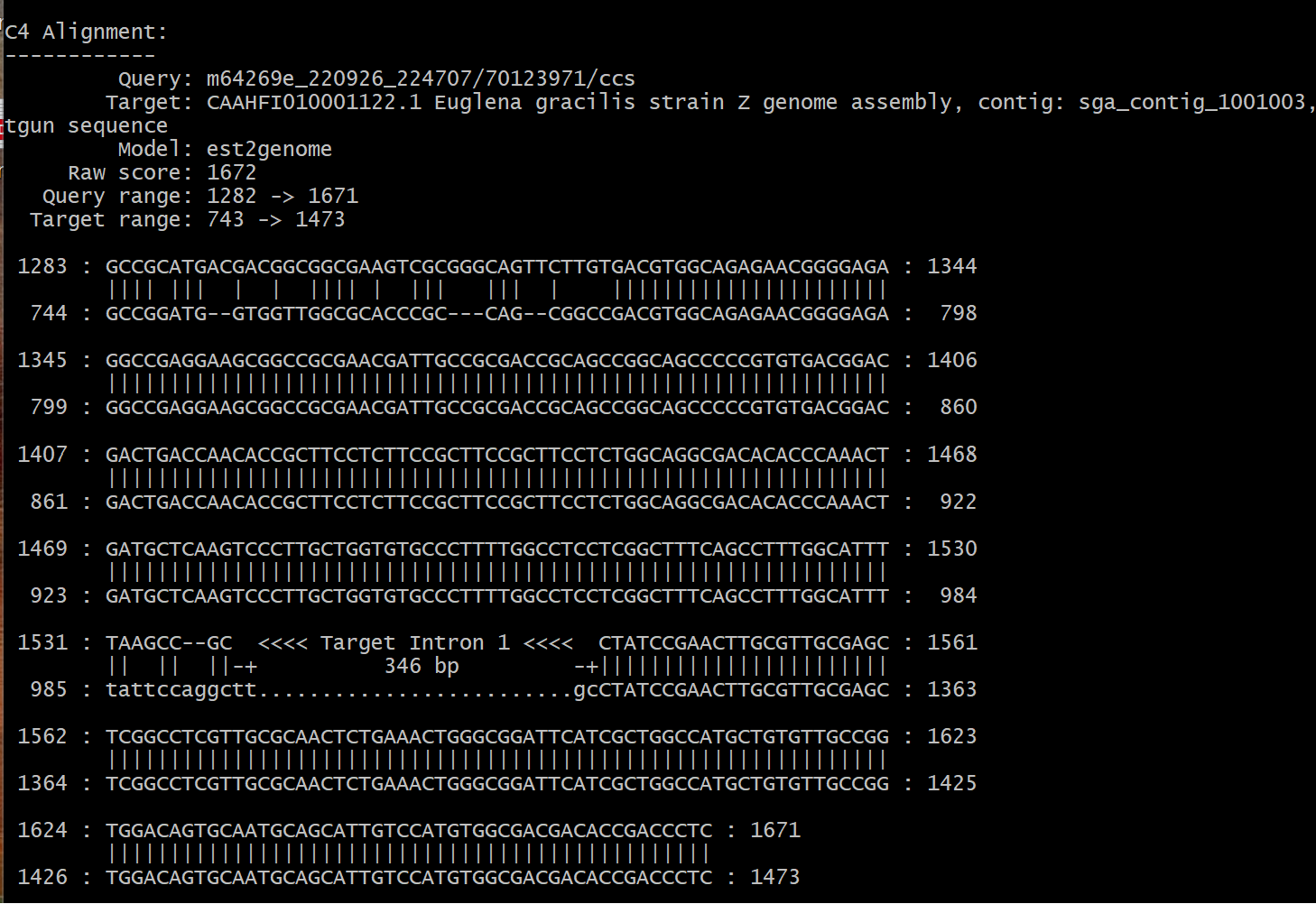


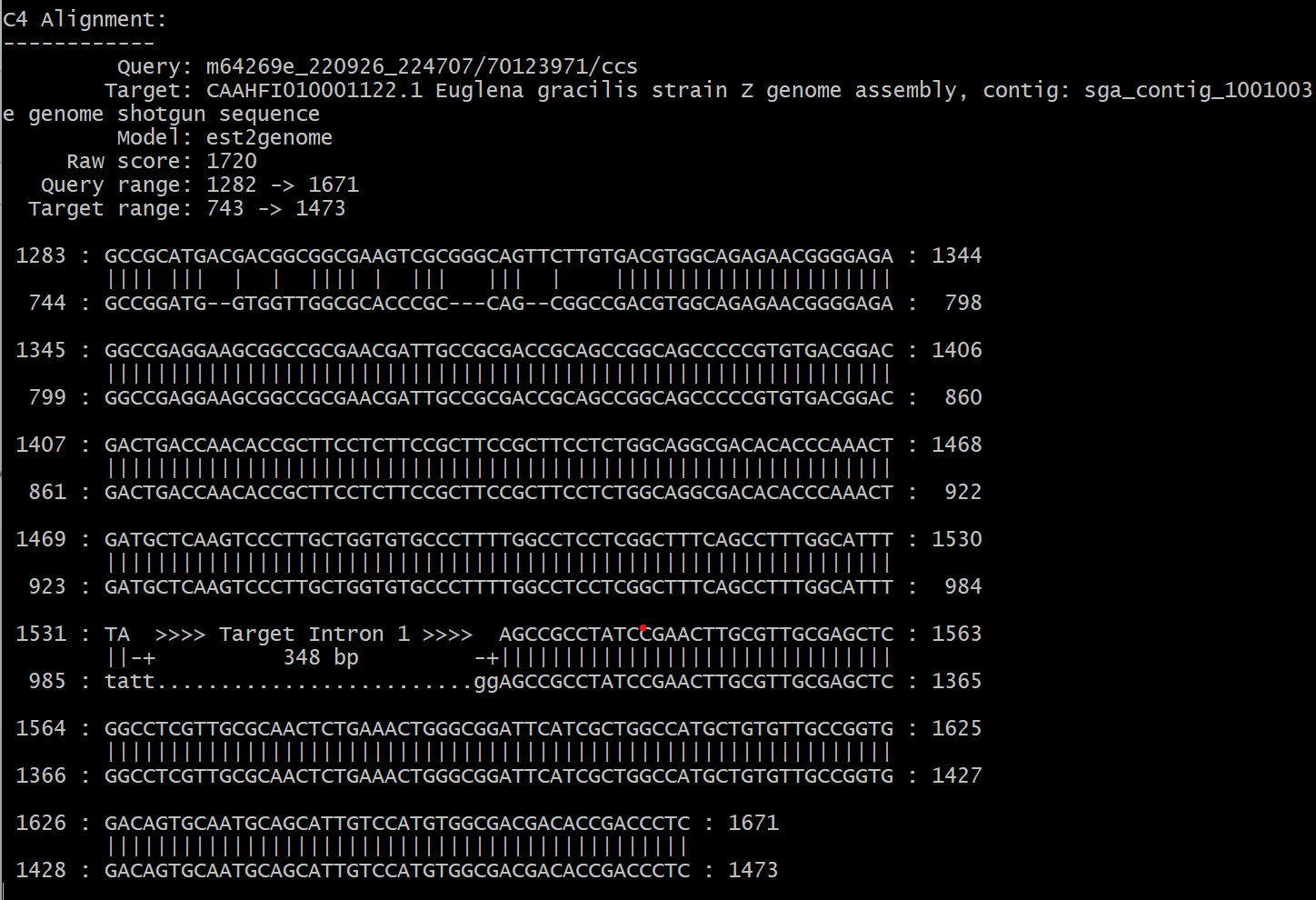


Supplementary Figure S1. Example of annotation of a nonconventional intron by the exonerate program with default (upper panel) and a custom PSSM (low panel).


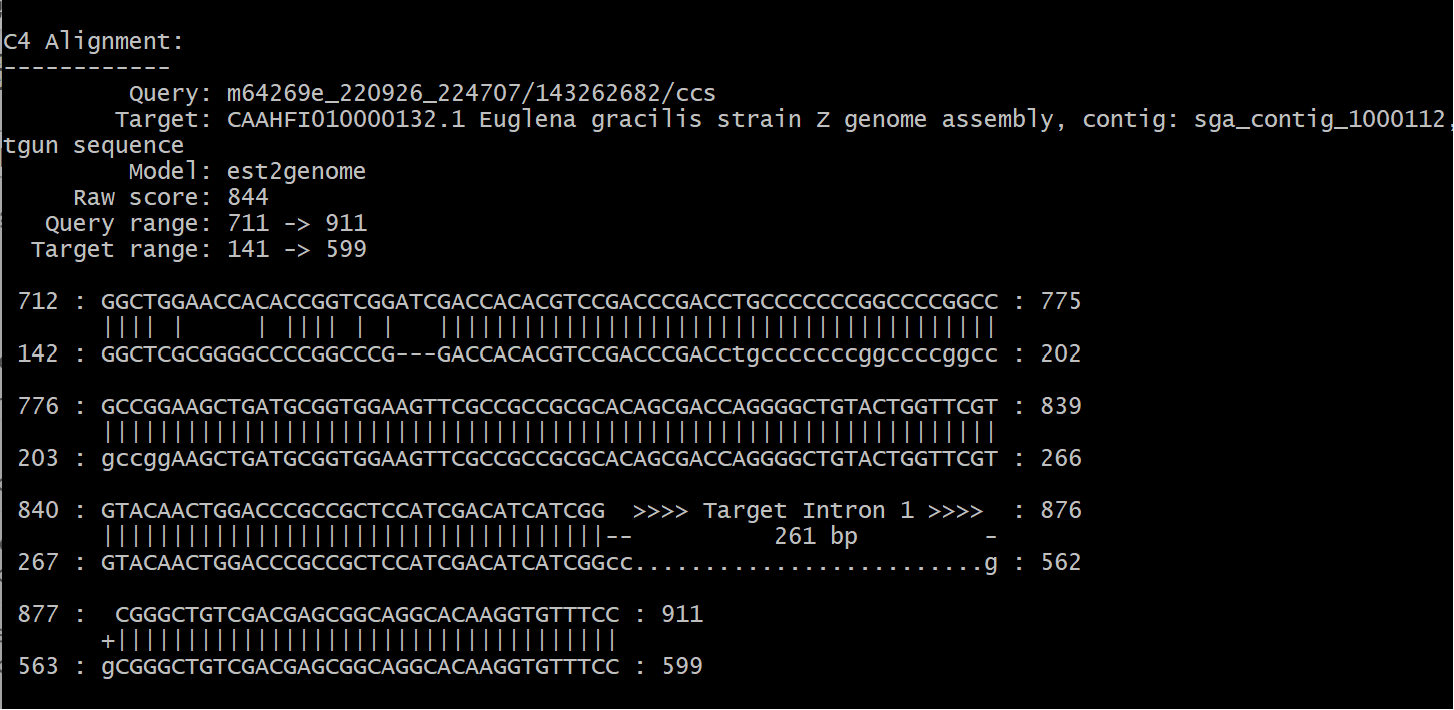


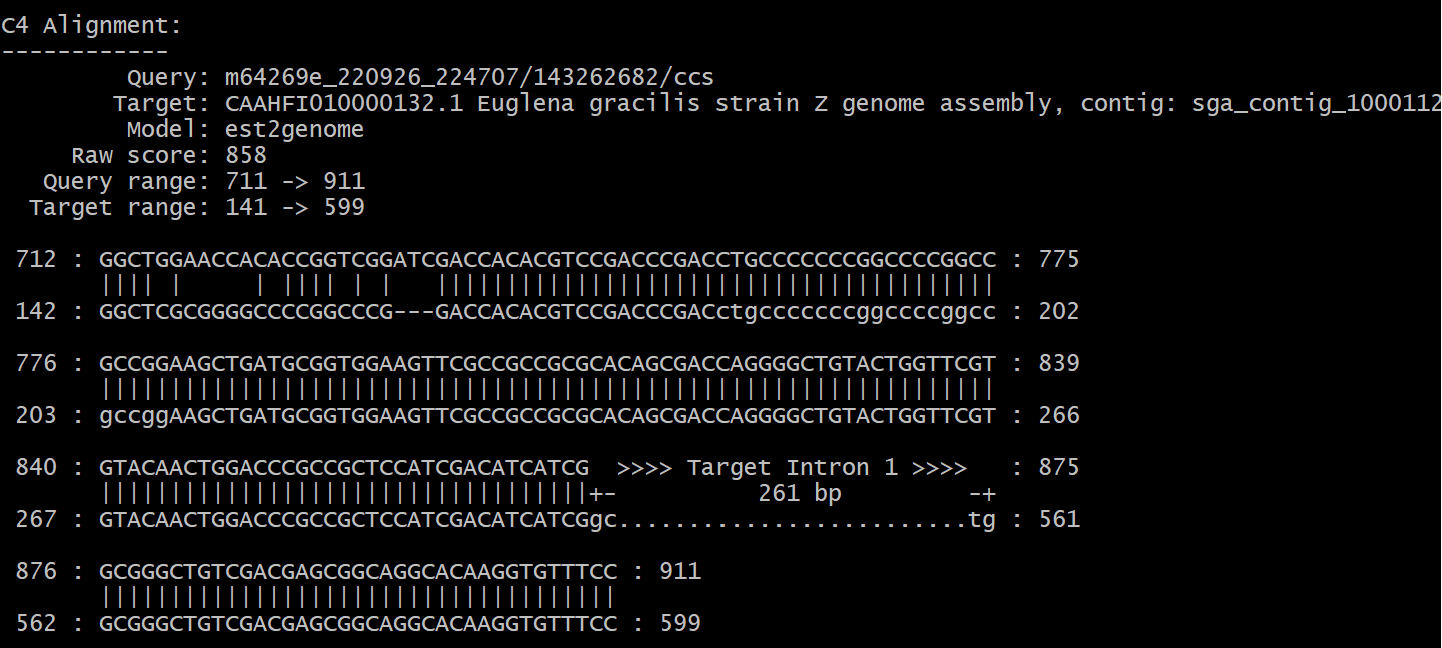


Supplementary Figure S2. Example of a ss-variant intron. Terminal nucleotides annotated by the exonerate program can be cc-gg (upper panel) or gc-tg (lower panel).


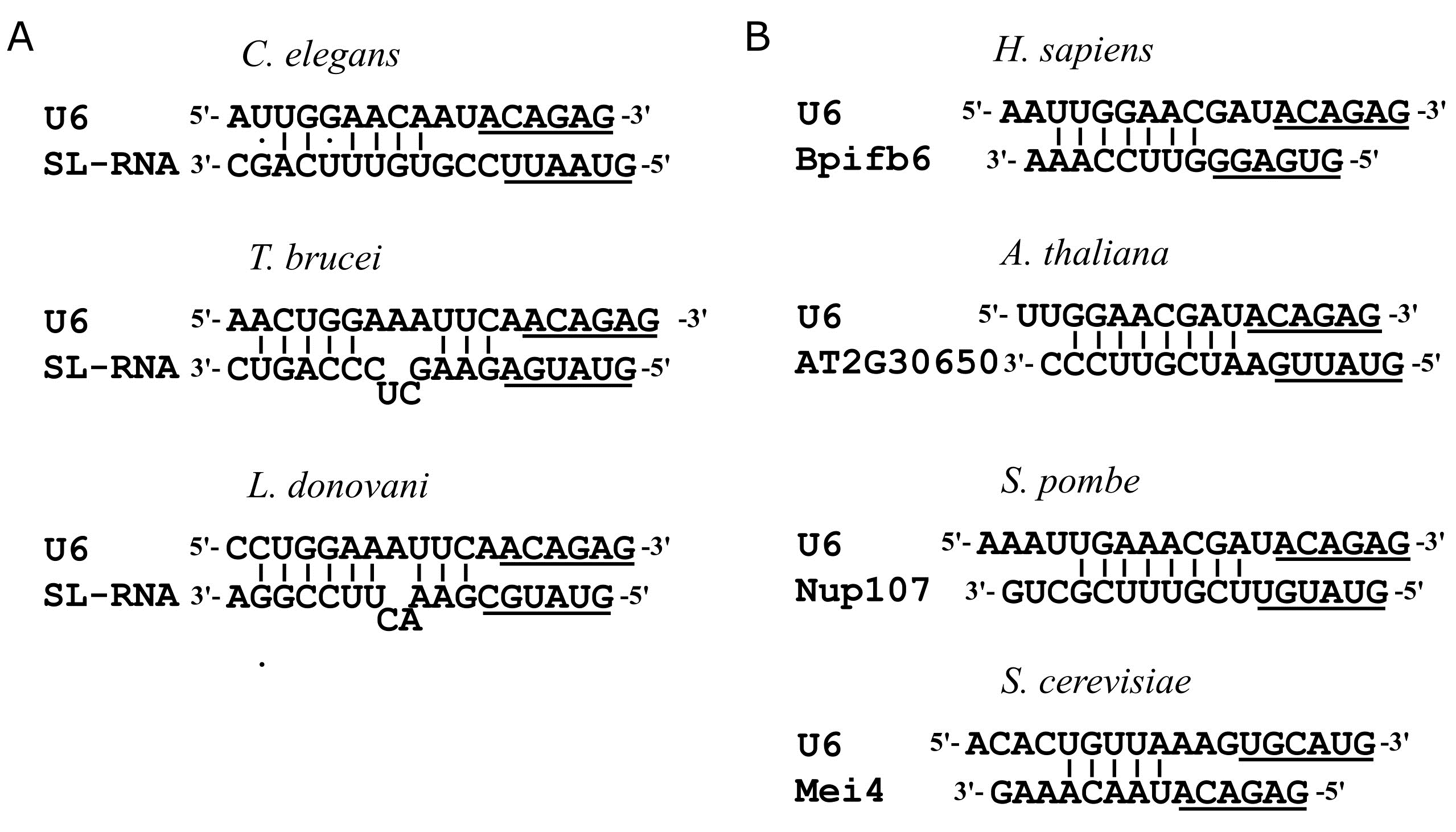


Supplementary Figure S3. The extended U6/5' ss helix in different species. (A) The extended U6/5' ss helix of SL-RNA in *C. elegans*, *T. brucei*, and *L. donovani*. (B) Examples of the extended U6/5' ss helix between the respective U6 and the 5' ss region of genes from *H. sapiens*, *A. thaliana*, *S. pombe*, and *S. cerevisiae*. The ACAGA box of U6 and the 5' ss of SL-RNA/intron are underlined.


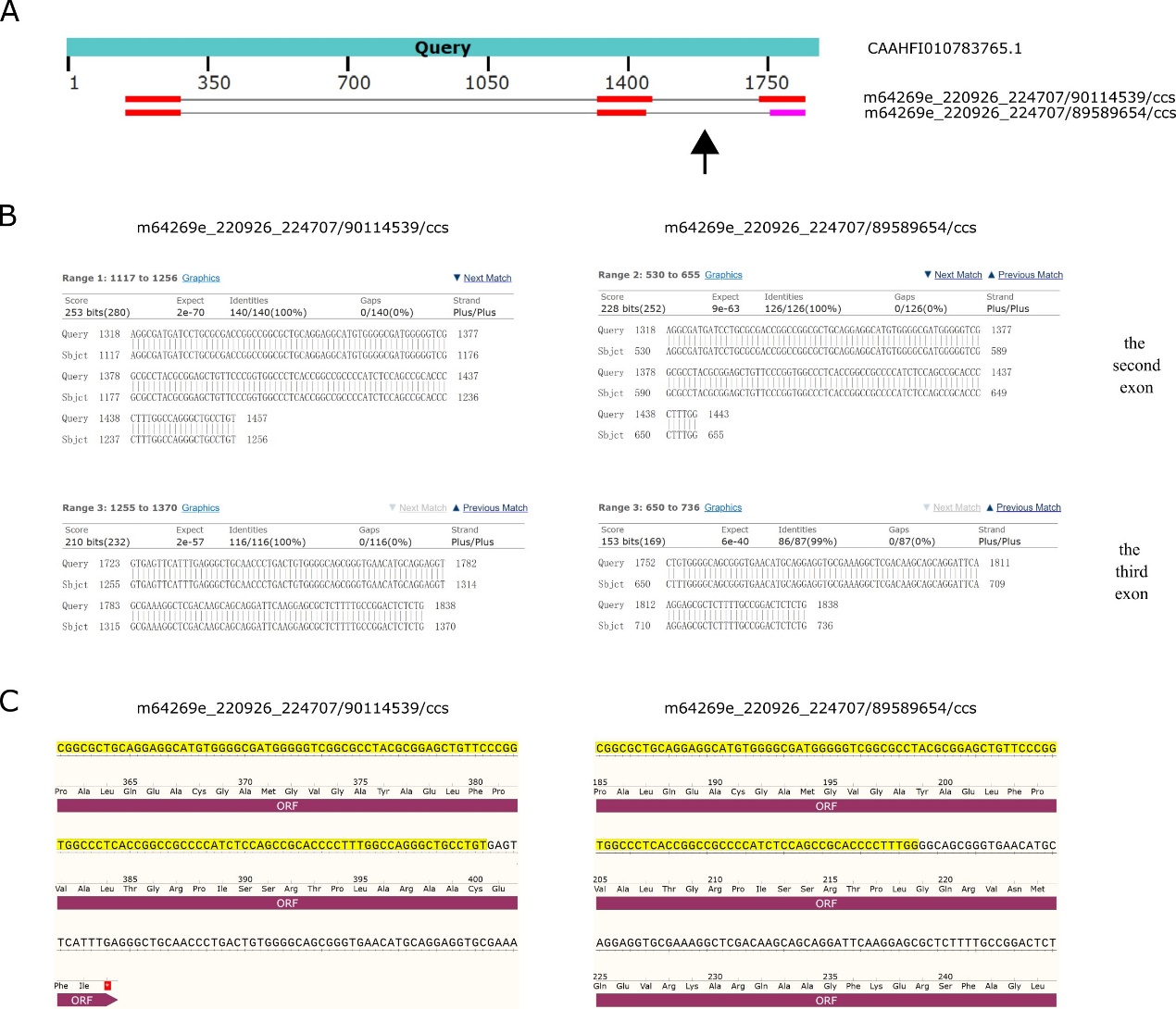


Supplementary Figure S4. An example of the AS pattern overlapping that affects the frameshift during translation. (A) Snapshot of the graphic summary of the sequence alignment generated by NCBI-BLAST program between the genomic DNA (used as query) and two RNA transcripts (used as subject) of a homolog of aarF domain-containing protein kinase 1 in *E. gracilis*. For the two transcripts, the second intron (indicated with a black arrow) between the second and the third exons is subject to overlapping. The length of this intron of the upper transcript (m64269e_220926_224707/90114539/ccs) is 267 bp and that of the lower transcript (m64269e_220926_224707/89589654/ccs) is 314 bp. (B) sequence alignment between the genomic DNA sequence and the second (upper panel) or third (lower panel) exons of the respective transcripts. (C) Translational frameshift of transcript m64269e_220926_224707/90114539/ccs in comparison to transcript m64269e_220926_224707/89589654/ccs. The second exons of both transcripts are colored in the yellow background. After intron removal, a stop codon occurs 9 nts downstream of this exon in m64269e_220926_224707/90114539/ccs.

Supplementary Table S1 AS events identified in this work

| AS type | Genomic accession | Transcriptomic accession | intron size (bp) |
| --- | --- | --- | --- |
| Overlapping | CAAHFI010095568.1 | m64269e_220926_224707/43059924/ccs  m64269e_220926_224707/47121973/ccs | 507  422 |
|  | CAAHFI010245853.1 | m64269e_220926_224707/39389135/ccs  m64269e_220926_224707/105775799/ccs | 244  181 |
|  | CAAHFI010365539.1 | m64269e_220926_224707/130025364/ccs  m64269e_220926_224707/24773118/ccs | 1275  1214 |
|  | CAAHFI010548366.1 | m64269e_220926_224707/38732698/ccs  m64269e_220926_224707/146606680/ccs | 1056  1001 |
|  | CAAHFI011060464.1 | m64269e_220926_224707/106299553/ccs  m64269e_220926_224707/77400317/ccs | 455  428 |
|  | CAAHFI011991134.1 | m64269e_220926_224707/59966605/ccs  m64269e_220926_224707/49941311/ccs | 632  586 |
|  | CAAHFI010356404.1 | m64269e_220926_224707/132121167/ccs  m64269e_220926_224707/98501514/ccs | 425  382 |
|  | CAAHFI010783765.1 | m64269e_220926_224707/89589654/ccs  m64269e_220926_224707/90114539/ccs | 314  267 |
|  | CAAHFI011214082.1 | m64269e_220926_224707/79364138/ccs  m64269e_220926_224707/151063201/ccs | 606  538 |
|  | CAAHFI011234586.1 | m64269e_220926_224707/5636690/ccs  m64269e_220926_224707/19858408/ccs | 626  509 |
|  | CAAHFI011313882.1 | m64269e_220926_224707/90245730/ccs  m64269e_220926_224707/45351257/ccs | 75  53 |
|  | CAAHFI011433261.1 | m64269e_220926_224707/55182940/ccs  m64269e_220926_224707/138610068/ccs | 361  325 |
|  | CAAHFI011569944.1 | m64269e_220926_224707/70126275/ccs  m64269e_220926_224707/86245980/ccs | 609  443 |
|  | CAAHFI011781967.1 | m64269e_220926_224707/127208210/ccs  m64269e_220926_224707/147130438/ccs | 257  252 |
|  | CAAHFI011989906.1 | m64269e_220926_224707/11797629/ccs  m64269e_220926_224707/156763211/ccs | 351  322 |
|  | CAAHFI012022742.1 | m64269e_220926_224707/56822410/ccs  m64269e_220926_224707/132317800/ccs | 569  547 |
|  | CAAHFI012050753.1 | m64269e_220926_224707/143066356/ccs  m64269e_220926_224707/139527452/ccs | 416  313 |
|  | CAAHFI011464222.1 | m64269e_220926_224707/79627380/ccs  m64269e_220926_224707/35258559/ccs | 625  505 |
|  | CAAHFI010081492.1 | m64269e_220926_224707/47974732/ccs  m64269e_220926_224707/124387789/ccs | 1189  1173 |
|  |  | m64269e_220926_224707/124387789/ccs  m64269e_220926_224707/2752992/ccs | 376  181 |
|  |  | m64269e_220926_224707/2752992/ccs  m64269e_220926_224707/124387789/ccs | 203  197 |
|  |  | m64269e_220926_224707/124387789/ccs  m64269e_220926_224707/2752992/ccs | 1173  927 |
|  | CAAHFI011326790.1 | m64269e_220926_224707/156304278/ccs  m64269e_220926_224707/119079977/ccs | 539  490 |
|  | CAAHFI011718962.1 | m64269e_220926_224707/100993234/ccs  m64269e_220926_224707/1705157/ccs | 2481  616 |
|  | CAAHFI010851876.1 | m64269e_220926_224707/67504620/ccs  m64269e_220926_224707/31393641/ccs | 464  428 |
|  |  | m64269e_220926_224707/67504620/ccs  m64269e_220926_224707/31393641/ccs | 698  691 |
|  | CAAHFI010347071.1 | m64269e_220926_224707/48498241/ccs  m64269e_220926_224707/170526546/ccs | 643  631 |
|  | CAAHFI010347071.1 | m64269e_220926_224707/76612282/ccs  m64269e_220926_224707/170526546/ccs | 620  631 |
|  | CAAHFI010521295.1 | m64269e_220926_224707/163187180/ccs  m64269e_220926_224707/49414239/ccs | 890  853 |
|  | CAAHFI010536519.1 | m64269e_220926_224707/165676250/ccs  m64269e_220926_224707/30869908/ccs | 731  708 |
|  | CAAHFI010572383.1 | m64269e_220926_224707/72223500/ccs  m64269e_220926_224707/170198887/ccs | 384  366 |
|  | CAAHFI010909693.1 | m64269e_220926_224707/155649477/ccs  m64269e_220926_224707/65864500/ccs | 820  764 |
|  | CAAHFI010959876.1 | m64269e_220926_224707/96536236/ccs  m64269e_220926_224707/146342703/ccs | 282  181 |
|  | CAAHFI011183243.1 | m64269e_220926_224707/52626203/ccs  m64269e_220926_224707/85983447/ccs | 226  182 |
|  | CAAHFI011276374.1 | m64269e_220926_224707/176882287/ccs  m64269e_220926_224707/114819535/ccs | 537  476 |
|  | CAAHFI011317307.1 | m64269e_220926_224707/55249255/ccs  m64269e_220926_224707/2883957/ccs | 431  405 |
|  | CAAHFI011342166.1 | m64269e_220926_224707/50397362/ccs  m64269e_220926_224707/177537392/ccs | 661  592 |
|  | CAAHFI011552157.1 | m64269e_220926_224707/11666435/ccs  m64269e_220926_224707/136907271/ccs | 643  633 |
|  | CAAHFI011557255.1 | m64269e_220926_224707/92473232/ccs  m64269e_220926_224707/14024974/ccs | 1125  976 |
|  | CAAHFI011902270.1 | m64269e_220926_224707/133366989/ccs  m64269e_220926_224707/170853083/ccs | 453  332 |
|  | CAAHFI011958562.1 | m64269e_220926_224707/122815136/ccs  m64269e_220926_224707/151192962/ccs | 186  155 |
|  | CAAHFI011972143.1 | m64269e_220926_224707/54003200/ccs  m64269e_220926_224707/110166862/ccs | 424  176 |
|  | CAAHFI011991608.1 | m64269e_220926_224707/92405916/ccs  m64269e_220926_224707/163184746/ccs | 197  174 |
|  | CAAHFI010295473.1 | m64269e_220926_224707/92602803/ccs  m64269e_220926_224707/152897144/ccs | 1534  1528 |
|  | CAAHFI010546828.1 | m64269e_220926_224707/28575217/ccs  m64269e_220926_224707/123995553/ccs | 797  650 |
|  | CAAHFI010580339.1 | m64269e_220926_224707/44630174/ccs  m64269e_220926_224707/21233997/ccs | 401  354 |
|  | CAAHFI010667019.1 | m64269e_220926_224707/128647318/ccs  m64269e_220926_224707/170590327/ccs | 383  244 |
|  | CAAHFI010874038.1 | m64269e_220926_224707/25561424/ccs  m64269e_220926_224707/25495777/ccs | 650  571 |
|  | CAAHFI011119898.1 | m64269e_220926_224707/61344480/ccs  m64269e_220926_224707/108266360/ccs | 529  490 |
|  | CAAHFI011660197.1 | m64269e_220926_224707/119932389/ccs  m64269e_220926_224707/118947893/ccs | 587  578 |
|  | CAAHFI012023185.1 | m64269e_220926_224707/15991524/ccs  m64269e_220926_224707/6817316/ccs | 891  855 |
|  | CAAHFI012051415.1 | m64269e_220926_224707/113770944/ccs  m64269e_220926_224707/123668601/ccs | 317  296 |
|  | CAAHFI010042953.1 | m64269e_220926_224707/25822647/ccs  m64269e_220926_224707/84149969/ccs | 168  107 |
|  | CAAHFI010272689.1 | m64269e_220926_224707/152371829/ccs  m64269e_220926_224707/87755449/ccs | 983  976 |
|  | CAAHFI010699269.1 | m64269e_220926_224707/168626511/ccs  m64269e_220926_224707/41158690/ccs | 1489  1416 |
|  | CAAHFI011451322.1 | m64269e_220926_224707/152764890/ccs  m64269e_220926_224707/117245561/ccs | 1338  1302 |
|  | CAAHFI011883343.1 | m64269e_220926_224707/85131491/ccs  m64269e_220926_224707/172753631/ccs | 611  580 |
|  | CAAHFI011008416.1 | m64269e_220926_224707/116327091  m64269e_220926_224707/138085842 | 607  595 |
|  | CAAHFI011655251.1 | m64269e_220926_224707/64029565/ccs  m64269e_220926_224707/60490157/ccs | 723  696 |
| A5SS | CAAHFI011091074.1 | m64269e_220926_224707/127140882/ccs  m64269e_220926_224707/57018893/ccs | 473  422 |
|  | CAAHFI010422330.1 | m64269e_220926_224707/134284305/ccs  m64269e_220926_224707/5046787/ccs | 308  263 |
|  | CAAHFI010306310.1 | m64269e_220926_224707/102236931/ccs  m64269e_220926_224707/88605166/ccs | 213  189 |
|  | CAAHFI011051963.1 | m64269e_220926_224707/155910693/ccs  m64269e_220926_224707/85721392/ccs | 1898  1860 |
|  | CAAHFI011442426.1 | m64269e_220926_224707/114558393/ccs  m64269e_220926_224707/85002576/ccs | 657  586 |
|  | CAAHFI010347071.1 | m64269e_220926_224707/48498241/ccs  m64269e_220926_224707/76612282/ccs | 643  620 |
|  | CAAHFI010354116.1 | m64269e_220926_224707/29820316/ccs  m64269e_220926_224707/93782689/ccs | 417  409 |
|  | CAAHFI010388402.1 | m64269e_220926_224707/7013374/ccs  m64269e_220926_224707/117573555/ccs | 265  228 |
|  | CAAHFI011223848.1 | m64269e_220926_224707/170460429/ccs  m64269e_220926_224707/28117570/ccs | 1010  1005 |
| A3SS | CAAHFI011004980.1 | m64269e_220926_224707/149948060/ccs  m64269e_220926_224707/34604746/ccs | 1468  1406 |
|  | CAAHFI011779575.1 | m64269e_220926_224707/156239323/ccs  m64269e_220926_224707/92865199/ccs | 814  745 |
|  | CAAHFI011133615.1 | m64269e_220926_224707/56297830/ccs  m64269e_220926_224707/2032067/ccs | 3515  3454 |
|  | CAAHFI010851876.1 | m64269e_220926_224707/67504620/ccs  m64269e_220926_224707/31393641/ccs | 400  376 |
|  | CAAHFI010708712.1 | m64269e_220926_224707/23726551/ccs  m64269e_220926_224707/24316759/ccs | 1421  1368 |
|  | CAAHFI010727729.1 | m64269e_220926_224707/14092929/ccs  m64269e_220926_224707/149554050/ccs | 594  591 |
|  | CAAHFI010793419.1 | m64269e_220926_224707/167641783/ccs  m64269e_220926_224707/134743117/ccs | 962  950 |
|  | CAAHFI011631804.1 | m64269e_220926_224707/46202977/ccs  m64269e_220926_224707/171377062/ccs | 259  181 |
|  | CAAHFI011819729.1 | m64269e_220926_224707/72745495/ccs  m64269e_220926_224707/111609248/ccs | 544  499 |
| SE | CAAHFI010928795.1 | m64269e_220926_224707/6947845/ccs  m64269e_220926_224707/73729733/ccs | 313  157/90 |
| atypical SE | CAAHFI010816564.1 | m64269e_220926_224707/124977878/ccs  m64269e_220926_224707/91359879/ccs | 740  149/568 |
|  | CAAHFI010746424.1 | m64269e_220926_224707/92539151/ccs  m64269e_220926_224707/107611860/ccs | 1591  172/52/400/395/128 |
|  | CAAHFI010851876.1 | m64269e_220926_224707/31393641/ccs  m64269e_220926_224707/67504620/ccs | 901  337/310 |
|  | CAAHFI010002354.1 | m64269e_220926_224707/103154417/ccs  m64269e_220926_224707/6032004/ccs | 601  389/111 |
|  | CAAHFI010884710.1* | m64269e_220926_224707/157615496/ccs  m64269e_220926_224707/72157398/ccs | 2546  1184/1293 |
|  | CAAHFI010971903.1* | m64269e_220926_224707/131924688/ccs  m64269e_220926_224707/67635903/ccs | 943  225/563 |
|  | CAAHFI011136820.1 | m64269e_220926_224707/3145862/ccs  m64269e_220926_224707/71631551/ccs | 784  47/422 |
|  | CAAHFI011903693.1 | m64269e_220926_224707/69338870/ccs  m64269e_220926_224707/176293414/ccs | 2498  1818/510 |
|  | CAAHFI010206979.1 | m64269e_220926_224707/165675916/ccs  m64269e_220926_224707/58392993/ccs | 2066  1216/864 |
|  | CAAHFI010129350.1* | m64269e_220926_224707/120063365/ccs  m64269e_220926_224707/53609876/ccs | 850  89/728 |
| RI | CAAHFI010251568.1 | m64269e_220926_224707/124453184/ccs  m64269e_220926_224707/52560265/ccs | 301 |
|  | CAAHFI010251568.1 | m64269e_220926_224707/124453184/ccs  m64269e_220926_224707/52560265/ccs | 569 |
|  | CAAHFI010183057.1 | m64269e_220926_224707/131599195/ccs  m64269e_220926_224707/46204556/ccs | 206 |
|  | CAAHFI010117114.1 | m64269e_220926_224707/178652730/ccs  m64269e_220926_224707/25756321/ccs | 301 |
|  | CAAHFI010252207.1 | m64269e_220926_224707/9240933/ccs  m64269e_220926_224707/127861445/ccs | 107 |
|  | CAAHFI010424696.1 | m64269e_220926_224707/119799873/ccs  m64269e_220926_224707/21301410/ccs | 427 |
|  | CAAHFI010424696.1 | m64269e_220926_224707/119799873/ccs  m64269e_220926_224707/21301410/ccs | 64 |
|  | CAAHFI010458001.1 | m64269e_220926_224707/170657313/ccs  64269e_220926_224707/83427732/ccs | 46 |
|  | CAAHFI011181616.1 | m64269e_220926_224707/176423776/ccs  m64269e_220926_224707/163447644/ccs | 43 |
|  | CAAHFI011268395.1 | m64269e_220926_224707/44763529/ccs  m64269e_220926_224707/162267615/ccs | 38 |
|  | CAAHFI011313882.1 | m64269e_220926_224707/45351257/ccs  m64269e_220926_224707/76546619/ccs | 53/75 |
|  | CAAHFI011342166.1 | m64269e_220926_224707/177537392/ccs  m64269e_220926_224707/50397362/ccs | 75 |
|  | CAAHFI011441990.1/CAAHFI010008997.1 | m64269e_220926_224707/75499924/ccs  m64269e_220926_224707/173146546/ccs | 459 |
|  | CAAHFI011445760.1/CAAHFI011768082.1 | m64269e_220926_224707/86181388/ccs  m64269e_220926_224707/17498667/ccs | 323 |
|  | CAAHFI010834601.1 | m64269e_220926_224707/9240633/ccs  m64269e_220926_224707/81331924/ccs  m64269e_220926_224707/49939395/ccs | 137/202/256/639/186 |
|  | CAAHFI010840030.1/CAAHFI010871724.1 | m64269e_220926_224707/105908210/ccs  m64269e_220926_224707/47710286/ccs | 257 |
|  | CAAHFI010910719.1 | m64269e_220926_224707/33095776/ccs  m64269e_220926_224707/99616075/ccs | 943 |
|  | CAAHFI010938757.1 | m64269e_220926_224707/69143116/ccs  m64269e_220926_224707/8323678/ccs | 106 |
|  | CAAHFI011136523.1 | m64269e_220926_224707/57149828/ccs  m64269e_220926_224707/28379574/ccs | 268 |
|  | CAAHFI011136523.1 | m64269e_220926_224707/57149828/ccs  m64269e_220926_224707/28379574/ccs | 301 |
|  | CAAHFI011136523.1 | m64269e_220926_224707/57149828/ccs  m64269e_220926_224707/28379574/ccs | 317 |
|  | CAAHFI011212125.1 | m64269e_220926_224707/95552345/ccs  m64269e_220926_224707/3672563/ccs | 527 |
|  | CAAHFI011212125.1 | m64269e_220926_224707/95552345/ccs  m64269e_220926_224707/3672563/ccs | 533 |
|  | CAAHFI011248382.1 | m64269e_220926_224707/36504281/ccs  m64269e_220926_224707/105253532/ccs | 276 |
|  | CAAHFI011381915.1 | m64269e_220926_224707/6096316/ccs  64269e_220926_224707/59116066/ccs | 245 |
|  | CAAHFI011894065.1 | m64269e_220926_224707/107874445/ccs  m64269e_220926_224707/48563120/ccs | 47 |
|  | CAAHFI011894065.1 | m64269e_220926_224707/107874445/ccs  m64269e_220926_224707/48563120/ccs | 45 |
|  | CAAHFI011956605.1/CAAHFI0111787/87.1 | m64269e_220926_224707/32704569/ccs  64269e_220926_224707/129500553/ccs | 443 |
|  | CAAHFI011958562.1 | m64269e_220926_224707/151192962/ccs  m64269e_220926_224707/122815136/ccs  m64269e_220926_224707/97780953/ccs | 299/186/155 |
|  | CAAHFI010089739.1 | m64269e_220926_224707/18088509/ccs m64269e_220926_224707/23986587/ccs | 52 |
|  | CAAHFI010287594.1 | m64269e_220926_224707/19007685/ccs m64269e_220926_224707/123472344/ccs | 146 |
|  | CAAHFI010289419.1 | m64269e_220926_224707/64553644/ccs  m64269e_220926_224707/132317639/ccs | 86 |
|  | CAAHFI010545152.1 | m64269e_220926_224707/22675755/ccs  m64269e_220926_224707/99485865/ccs | 657 |
|  | CAAHFI010553483.1/CAAHFI011315372.1 | m64269e_220926_224707/47580562/ccs  m64269e_220926_224707/53871888/ccs | 774 |
|  | CAAHFI010556605.1 | m64269e_220926_224707/88935172/ccs  m64269e_220926_224707/72288817/ccs | 366 |
|  | CAAHFI010621152.1 | m64269e_220926_224707/61802380/ccs  m64269e_220926_224707/99879018/ccs | 206/53 |
|  | CAAHFI010642553.1 | m64269e_220926_224707/29690618/ccs  m64269e_220926_224707/55246883/ccs | 423 |
|  | CAAHFI010739965.1 | m64269e_220926_224707/2361516/ccs  64269e_220926_224707/97518047/ccs | 384 |
|  | CAAHFI010777982.1 | m64269e_220926_224707/74711382/ccs  m64269e_220926_224707/34015495/ccs | 492 |
|  | CAAHFI010798983.1 | m64269e_220926_224707/140641993/ccs  m64269e_220926_224707/77923112/ccs | 344/60 |
|  | CAAHFI010808994.1 | m64269e_220926_224707/94109738/ccs  m64269e_220926_224707/142936872/ccs | 86 |
|  | CAAHFI010867420.1 | m64269e_220926_224707/112984650/ccs  m64269e_220926_224707/90767712/ccs | 44 |
|  | CAAHFI010876927.1 | m64269e_220926_224707/56494121/ccs  m64269e_220926_224707/24447304/ccs | 161 |
|  | CAAHFI010997633.1 | m64269e_220926_224707/140904706/ccs  m64269e_220926_224707/53674879/ccs | 46 |
|  | CAAHFI011003871.1 | m64269e_220926_224707/86902153/ccs  m64269e_220926_224707/37486700/ccs | 46 |
|  | CAAHFI011055188.1 | m64269e_220926_224707/166724564/ccs  m64269e_220926_224707/131205235/ccs | 187 |
|  | CAAHFI011058993.1 | m64269e_220926_224707/109512973/ccs  m64269e_220926_224707/108857003/ccs | 371 |
|  | CAAHFI011205260.1 | m64269e_220926_224707/72352247/ccs  m64269e_220926_224707/176424398/ccs | 79 |
|  | CAAHFI011336853.1 | m64269e_220926_224707/131402195/ccs  m64269e_220926_224707/82182908/ccs | 802 |
|  | CAAHFI011375181.1 | m64269e_220926_224707/104333554/ccs  m64269e_220926_224707/125239935/ccs | 698 |
|  | CAAHFI011461811.1 | m64269e_220926_224707/87820527/ccs  m64269e_220926_224707/157878403/ccs | 109 |
|  | CAAHFI011519620.1 | m64269e_220926_224707/68225104/ccs  m64269e_220926_224707/20056487/ccs | 119 |
|  | CAAHFI011533207.1 | m64269e_220926_224707/7537261/ccs  m64269e_220926_224707/45353457/ccs | 488 |
|  | CAAHFI011582030.1/CAAHFI011356103.1 | m64269e_220926_224707/77007101/ccs  m64269e_220926_224707/166724855/ccs | 88 |
|  | CAAHFI011588633.1 | m64269e_220926_224707/138412971/ccs  m64269e_220926_224707/17041535/ccs | 99 |
|  | CAAHFI011591745.1 | m64269e_220926_224707/64028913/ccs  m64269e_220926_224707/177997138/ccs | 367 |
|  | CAAHFI011648406.1 | m64269e_220926_224707/78119856/ccs  m64269e_220926_224707/60752672/ccs | 146 |
|  | CAAHFI011660600.1 | m64269e_220926_224707/37358141/ccs  m64269e_220926_224707/154928478/ccs | 320 |
|  | CAAHFI011662811.1 | m64269e_220926_224707/34408416/ccs  m64269e_220926_224707/154141428/ccs | 42 |
|  | CAAHFI011864412.1/CAAHFI011960032.1 | m64269e_220926_224707/98371702/ccs  m64269e_220926_224707/1705605/ccs | 272 |
|  | CAAHFI011974773.1 | m64269e_220926_224707/122686065/ccs  m64269e_220926_224707/144376982/ccs | 53/53 |
|  | CAAHFI012040318.1 | m64269e_220926_224707/163382336/ccs  m64269e_220926_224707/22022386/ccs | 92 |
|  | CAAHFI010015404.1 | m64269e_220926_224707/16975318/ccs  m64269e_220926_224707/34866553/ccs | 42 |
|  | CAAHFI010024059.1 | m64269e_220926_224707/94308925/ccs  m64269e_220926_224707/7800110/ccs | 256 |
|  | CAAHFI010042953.1 | m64269e_220926_224707/84149969/ccs  m64269e_220926_224707/88015560/ccs | 40 |
|  | CAAHFI010056306.1 | m64269e_220926_224707/94307933/ccs m64269e_220926_224707/151587211/ccs | 179 |
|  | CAAHFI010096060.1 | m64269e_220926_224707/175049399/ccs  m64269e_220926_224707/92997727/ccs | 49 |
|  | CAAHFI010103312.1 | m64269e_220926_224707/137953866/ccs  m64269e_220926_224707/57344423/ccs | 1080223/342 |
|  | CAAHFI010165720.1 | m64269e_220926_224707/8391098/ccs  m64269e_220926_224707/138479954/ccs | 50 |
|  | CAAHFI010175873.1 | m64269e_220926_224707/154599433/ccs  m64269e_220926_224707/7078780/ccs | 538/646 |
|  | CAAHFI010212341.1 | m64269e_220926_224707/140248245/ccs  m64269e_220926_224707/113903349/ccs | 273 |
|  | CAAHFI010221356.1/CAAHFI010806849.1 | m64269e_220926_224707/91817066/ccs  m64269e_220926_224707/87295368/ccs | 475 |
|  | CAAHFI010280974.1/CAAHFI010243923.1 | m64269e_220926_224707/79300653/ccs  m64269e_220926_224707/105581071/ccs | 138 |
|  | CAAHFI010305033.1 | m64269e_220926_224707/21102887/ccs  m64269e_220926_224707/116393009/ccs | 253/625 |
|  | CAAHFI010313717.1 | m64269e_220926_224707/151585502/ccs  m64269e_220926_224707/90966277/ccs | 120 |
|  | CAAHFI010365237.1 | m64269e_220926_224707/34537980/ccs  m64269e_220926_224707/99813463/ccs | 307 |
|  | CAAHFI010436661.1/CAAHFI010076854.1 | m64269e_220926_224707/16451610/ccs  m64269e_220926_224707/4129539/ccs | 267 |
|  | CAAHFI010477291.1 | m64269e_220926_224707/161744514/ccs  m64269e_220926_224707/124650129/ccs | 48 |
|  | CAAHFI010490763.1 | m64269e_220926_224707/81068160/ccs  m64269e_220926_224707/126484816/ccs | 85 |
|  | CAAHFI010561259.1 | m64269e_220926_224707/119802601/ccs  m64269e_220926_224707/14876856/ccs | 222 |
|  | CAAHFI010563531.1 | m64269e_220926_224707/98371573/ccs  m64269e_220926_224707/78907504/ccs | 325 |
|  | CAAHFI010580500.1 | m64269e_220926_224707/14551322/ccs  m64269e_220926_224707/158991364/ccs | 207/275 |
|  | CAAHFI010671002.1/CAAHFI011978759.1 | m64269e_220926_224707/38142104/ccs  m64269e_220926_224707/35782971/ccs | 127 |
|  | CAAHFI010740163.1 | m64269e_220926_224707/59639769/ccs m64269e_220926_224707/87950588/ccs | 69 |
|  | CAAHFI010790380.1 | m64269e_220926_224707/36373643/ccs m64269e_220926_224707/10683252/ccs | 43 |
|  | CAAHFI010808415.1 | m64269e_220926_224707/6947578/ccs  m64269e_220926_224707/23399160/ccs | 220 |
|  | CAAHFI010867865.1/CAAHFI011387466.1 | m64269e_220926_224707/29231513/ccs  m64269e_220926_224707/55118649/ccs | 104 |
|  | CAAHFI010979715.1 | m64269e_220926_224707/72615592/ccs  m64269e_220926_224707/120391163/ccs | 260/435 |
|  | CAAHFI011184642.1/CAAHFI011223267.1 | m64269e_220926_224707/106235146/ccs  m64269e_220926_224707/90704295/ccs | 49 |
|  | CAAHFI011251957.1 | m64269e_220926_224707/16976172/ccs  m64269e_220926_224707/80022139/ccs | 242/710 |
|  | CAAHFI011252142.1 | m64269e_220926_224707/72091012/ccs  m64269e_220926_224707/61669907/ccs | 45 |
|  | CAAHFI011339149.1 | m64269e_220926_224707/51317014/ccs  m64269e_220926_224707/167249750/ccs | 355 |
|  | CAAHFI011494914.1 | m64269e_220926_224707/1902432/ccs  m64269e_220926_224707/147652615/ccs | 347 |
|  | CAAHFI011514322.1 | m64269e_220926_224707/53740971/ccs  m64269e_220926_224707/167708422/ccs | 489 |
|  | CAAHFI011518668.1/CAAHFI011367957.1 | m64269e_220926_224707/12715887/ccs  m64269e_220926_224707/141363650/ccs | 736 |
|  | CAAHFI011548752.1/CAAHFI011985068.1 | m64269e_220926_224707/115279155/ccs  m64269e_220926_224707/93849977/ccs | 301 |
|  | CAAHFI011602900.1 | m64269e_220926_224707/165347735/ccs  m64269e_220926_224707/63309526/ccs | 126 |
|  | CAAHFI011658843.1 | m64269e_220926_224707/106824634/ccs  m64269e_220926_224707/37291225/ccs | 41 |
|  | CAAHFI011667060.1 | m64269e_220926_224707/50005958/ccs  m64269e_220926_224707/84084082/ccs | 609 |
|  | CAAHFI011682341.1/CAAHFI010052243.1 | m64269e_220926_224707/153946897/ccs  m64269e_220926_224707/105253299/ccs | 310 |
|  | CAAHFI011821328.1/CAAHFI010484113.1 | m64269e_220926_224707/23724442/ccs  m64269e_220926_224707/19989337/ccs | 666 |
|  | CAAHFI011857585.1 | m64269e_220926_224707/133498173/ccs  m64269e_220926_224707/127535178/ccs | 477 |
|  | CAAHFI011912623.1 | m64269e_220926_224707/29492184/ccs  m64269e_220926_224707/75302521/ccs | 237 |
|  | CAAHFI011917852.1/CAAHFI011586153.1 | m64269e_220926_224707/70912396/ccs  m64269e_220926_224707/45812438/ccs | 516 |
|  | CAAHFI011992067.1 | m64269e_220926_224707/117836412/ccs  m64269e_220926_224707/114885302/ccs | 46/48 |
|  | CAAHFI012034269.1/CAAHFI010098996.1 | m64269e_220926_224707/109774664/ccs  m64269e_220926_224707/18614675/ccs | 252 |
|  | CAAHFI010087310.1 | m64269e_220926_224707/33292576/ccs  m64269e_220926_224707/85984803/ccs | 44/51/51 |
|  | CAAHFI010798983.1 | m64269e_220926_224707/140641993/ccs  m64269e_220926_224707/77923112/ccs | 344/60 |
|  | CAAHFI010218620.1 | m64269e_220926_224707/27133920/ccs  m64269e_220926_224707/123929473/ccs | 102/237/75 |
|  | CAAHFI010746424.1 | m64269e_220926_224707/107611860/ccs  m64269e_220926_224707/124520885/ccs | 52 |
|  | CAAHFI011645292.1 | m64269e_220926_224707/42140649/ccs  m64269e_220926_224707/158467092/ccs | 174/318/598/95 |
|  | CAAHFI011439774.1 | m64269e_220926_224707/99812553/ccs  m64269e_220926_224707/102498909/ccs | 113/291/413/662 |
|  | CAAHFI010081492.1 | m64269e_220926_224707/2752992/ccs  m64269e_220926_224707/47974732/ccs | 927/181 |
|  | CAAHFI011947138.1 | m64269e_220926_224707/49939399/ccs  m64269e_220926_224707/97321100/ccs | 240/298 |
|  | CAAHFI010235868.1 | m64269e_220926_224707/158271152/ccs  m64269e_220926_224707/123471428/ccs | 493 |
|  | CAAHFI010248748.1 | m64269e_220926_224707/138807764/ccs m64269e_220926_224707/39977266/ccs | 191 |
|  | CAAHFI010546773.1/CAAHFI011462264.1 | m64269e_220926_224707/21433056/ccs m64269e_220926_224707/141101184/ccs | 47 |
|  | CAAHFI010552633.1 | m64269e_220926_224707/64883021/ccs  m64269e_220926_224707/175899233/ccs | 220 |
|  | CAAHFI010600833.1 | m64269e_220926_224707/89459215/ccs  m64269e_220926_224707/109971974/ccs | 977 |
|  | CAAHFI010617149.1 | m64269e_220926_224707/131138402/ccs  m64269e_220926_224707/175901328/ccs | 59/613 |
|  | CAAHFI010634071.1 | m64269e_220926_224707/9635362/ccs  m64269e_220926_224707/12453269/ccs | 440/64 |
|  | CAAHFI010694525.1/CAAHFI010443843.1 | m64269e_220926_224707/31260827/ccs  m64269e_220926_224707/13434903/ccs | 82 |
|  | CAAHFI010804878.1 | m64269e_220926_224707/132187969/ccs  m64269e_220926_224707/139855726/ccs | 143 |
|  | CAAHFI010959876.1 | m64269e_220926_224707/96536236/ccs  m64269e_220926_224707/51642752/ccs | 282/290/303 |
|  | CAAHFI010967293.1 | m64269e_220926_224707/32704569/ccs  m64269e_220926_224707/129500553/ccs | 443 |
|  | CAAHFI011055609.1 | m64269e_220926_224707/89721314/ccs m64269e_220926_224707/149292565/ccs | 132/66/909 |
|  | CAAHFI011367070.1 | m64269e_220926_224707/39519676/ccs m64269e_220926_224707/51905047/ccs | 512/626 |
|  | CAAHFI011408146.1 | m64269e_220926_224707/92078858/ccs  m64269e_220926_224707/133890605/ccs | 415 |
|  | CAAHFI011479801.1 | m64269e_220926_224707/78578814/ccs  m64269e_220926_224707/153946956/ccs | 55 |
|  | CAAHFI011510581.1 | m64269e_220926_224707/57018669/ccs  m64269e_220926_224707/95093244/ccs | 418 |
|  | CAAHFI011522833.1 | m64269e_220926_224707/177998240/ccs  m64269e_220926_224707/159581740/ccs | 660 |
|  | CAAHFI011635244.1 | m64269e_220926_224707/179899056/ccs  m64269e_220926_224707/129959467/ccs | 193 |
|  | CAAHFI011643931.1 | m64269e_220926_224707/127076143/ccs  m64269e_220926_224707/79563418/ccs | 187/632/669 |
|  | CAAHFI011734280.1 | m64269e_220926_224707/122619124/ccs  m64269e_220926_224707/168429499/ccs | 251/378/442 |
|  | CAAHFI011735749.1 | m64269e_220926_224707/129042828/ccs  m64269e_220926_224707/18088090/ccs | 80 |
|  | CAAHFI011806276.1 | m64269e_220926_224707/158335562/ccs  m64269e_220926_224707/100665998/ccs | 1326/98 |
|  | CAAHFI011923938.1/CAAHFI011097670.1 | m64269e_220926_224707/64226920/ccs  m64269e_220926_224707/165743134/ccs | 514 |
|  | CAAHFI011948404.1 | m64269e_220926_224707/133562940/ccs  m64269e_220926_224707/96731853/ccs | 115/143 |
|  | CAAHFI010072932.1 | m64269e_220926_224707/119081761/ccs  m64269e_220926_224707/67701420/ccs | 367 |
|  | CAAHFI010244548.1 | m64269e_220926_224707/116591088/ccs  m64269e_220926_224707/142477275/ccs | 107 |
|  | CAAHFI010645279.1 | m64269e_220926_224707/157158187/ccs  m64269e_220926_224707/45417719/ccs | 197 |
|  | CAAHFI010761963.1 | m64269e_220926_224707/167315204/ccs  m64269e_220926_224707/7145781/ccs | 46/47/47/48/53 |
|  | CAAHFI011156458.1 | m64269e_220926_224707/163055438/ccs  m64269e_220926_224707/67436738/ccs | 213/440/484/86 |
|  | CAAHFI011430762.1 | m64269e_220926_224707/179243393/ccs  m64269e_220926_224707/164955899/ccs | 228 |
|  | CAAHFI011584046.1 | m64269e_220926_224707/30737336/ccs  m64269e_220926_224707/22217391/ccs | 134/204 |
|  | CAAHFI011703139.1 | m64269e_220926_224707/133958119/ccs  m64269e_220926_224707/74843461/ccs | 111/197/803 |
|  | CAAHFI010054903.1 | m64269e_220926_224707/122489486/ccs  m64269e_220926_224707/88605595/ccs | 1196/185/47/75/82 |
|  | CAAHFI010078595.1 | m64269e_220926_224707/60688656/ccs  m64269e_220926_224707/170852933/ccs | 234 |
|  | CAAHFI010272689.1 | m64269e_220926_224707/87755449/ccs  m64269e_220926_224707/152371829/ccs | 121 |
|  | CAAHFI010359876.1 | m64269e_220926_224707/174721391/ccs  m64269e_220926_224707/31524226/ccs | 120/205/257/495 |
|  | CAAHFI010928795.1 | m64269e_220926_224707/73729733/ccs  m64269e_220926_224707/6947845/ccs  m64269e_220926_224707/70583704/ccs | 157/311/313/90 |
|  | CAAHFI011004469.1 | m64269e_220926_224707/116263023/ccs  m64269e_220926_224707/79431872/ccs | 254/313 |
|  | CAAHFI011124639.1 | m64269e_220926_224707/22544738/ccs  m64269e_220926_224707/22544738/ccs | 86 |
|  | CAAHFI011451106.1 | m64269e_220926_224707/148635675/ccs  m64269e_220926_224707/9243427/ccs | 263/303/825 |
|  | CAAHFI011495331.1 | m64269e_220926_224707/90899949/ccs  m64269e_220926_224707/68684069/ccs | 212/308 |
|  | CAAHFI011721723.1 | m64269e_220926_224707/7932098/ccs  m64269e_220926_224707/78250482/ccs | 109/341 |
|  | CAAHFI011918215.1/CAAHFI011876870.1 | m64269e_220926_224707/92210343/ccs  m64269e_220926_224707/40764103/ccs | 165 |
|  | CAAHFI011961491.1 | m64269e_220926_224707/10422564/ccs  m64269e_220926_224707/51315372/ccs | 185/502/580 |
|  | CAAHFI010086765.1 | m64269e_220926_224707/48038957/ccs  m64269e_220926_224707/138347703/ccs | 240 |
|  | CAAHFI010463498.1 | m64269e_220926_224707/107873713/ccs  m64269e_220926_224707/18940608/ccs | 1013/380/59/67 |
|  | CAAHFI011860170.1 | m64269e_220926_224707/57999749/ccs  m64269e_220926_224707/93651673/ccs | 546 |

*same 5' ss
